# Molecular and supported Ti(iii)-alkyls: efficient ethylene polymerization driven by the π-character of metal–carbon bonds and back donation from a singly occupied molecular orbital†‡

**DOI:** 10.1039/d0sc04436a

**Published:** 2020-11-11

**Authors:** Anton Ashuiev, Florian Allouche, Nino Wili, Keith Searles, Daniel Klose, Christophe Copéret, Gunnar Jeschke

**Affiliations:** Department of Chemistry and Applied Biosciences, ETH Zürich Vladimir Prelog Weg 1-5 CH-8093 Zürich Switzerland ccoperet@ethz.ch gunnar.jeschke@phys.chem.ethz.ch

## Abstract

While Ti(iii) alkyl species are the proposed active sites in Ziegler–Natta ethylene polymerization catalysts, the corresponding well-defined homogeneous catalysts are not known. We report that well-defined neutral β-diiminato Ti(iii) alkyl species, namely [Ti(nacnac)(CH_2_^*t*^Bu)_2_] and its alumina-grafted derivative [(Al_s_O)Ti(nacnac)(CH_2_^*t*^Bu)], are active towards ethylene polymerization at moderate pressures and temperatures and possess an electron configuration well-adapted to insertion of ethylene. Advanced EPR spectroscopy showed that ethylene insertion into a Ti(iii)–C bond takes place during polymerization from Ti(nacnac)(CH_2_^*t*^Bu)_2_. A combination of pulsed EPR spectroscopy and DFT calculations, based on a crystal structure of [Ti(nacnac)(CH_2_^*t*^Bu)_2_], enabled us to reveal details about the structure and electronic configurations of both molecular and surface-grafted species. For both compounds, the α-agostic C–H interaction, which involves the singly occupied molecular orbital, indicates a π character of the metal–carbon bond; this π character is enhanced upon ethylene coordination, leading to a nearly barrier-less C_2_H_4_ insertion into Ti(iii)–C bonds after this first step. During coordination, back donation from the SOMO to the π*(C_2_H_4_) occurs, leading to stabilization of π-ethylene complexes and to a significant lowering of the overall energy of the C_2_H_4_ insertion transition state. In d^1^ alkyl complexes, ethylene insertion follows an original “augmented” Cossee–Arlman mechanism that involves the delocalization of unpaired electrons between the SOMO, π*(C_2_H_4_) and σ*(Ti–C) in the transition state, which further favors ethylene insertion. All these factors facilitate ethylene polymerization on Ti(iii) neutral alkyl species and make d^1^ alkyl complexes potentially more effective polymerization catalysts than their d^0^ analogues.

## Abstract 2

Ti(iii) alkyl species polymerize ethylene *via* an original mechanism, which involves back donation to the π*(C_2_H_4_) and a delocalization of the unpaired electron in the transition state of C_2_H_4_ insertion into the partially alkylidenic Ti(iii)–C bond.

## Graphical abstract

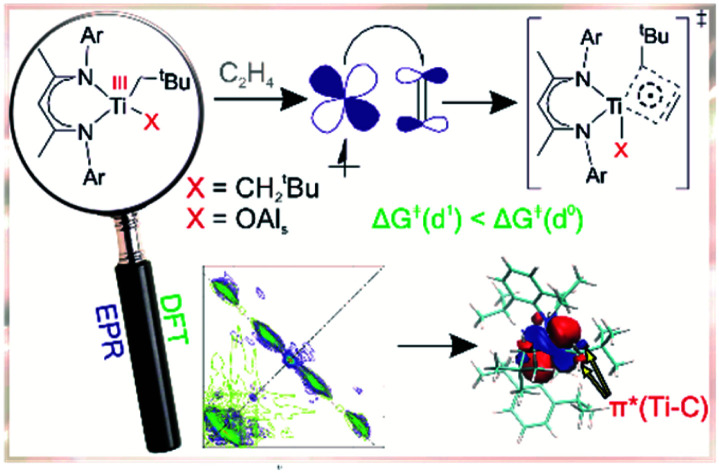

## Introduction

**Scheme 1:**
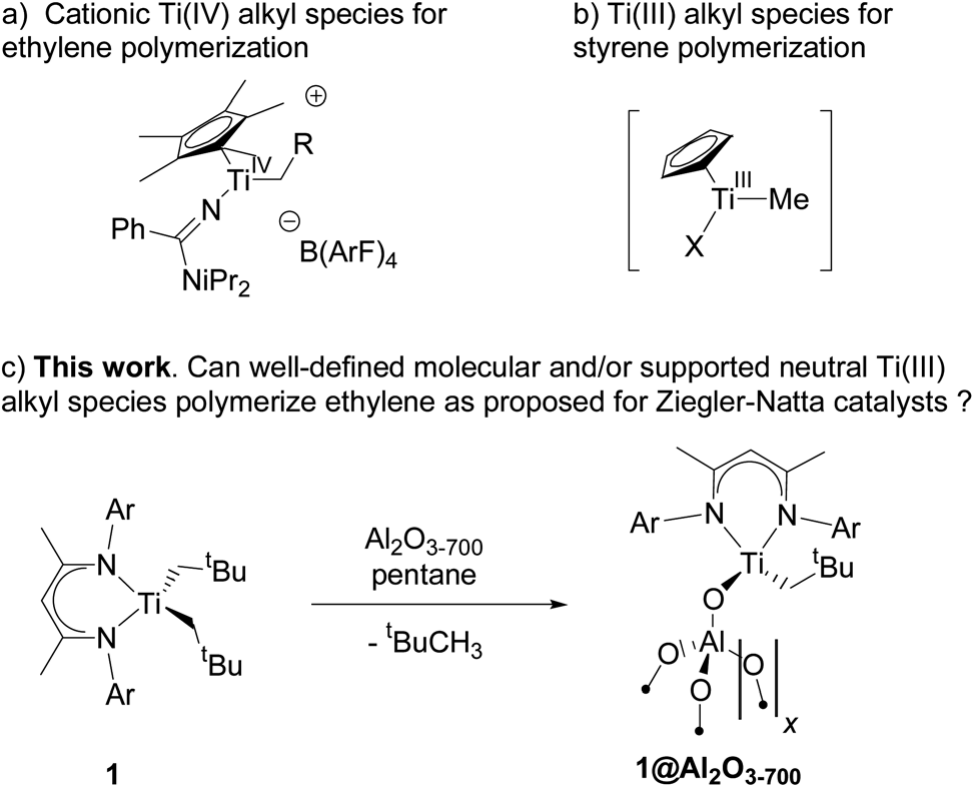
Known Ti(iv) (a) and Ti(iii) (b) alkyl species, competent in olefin polymerization, together with synthesis of well-defined neutral supported Ti(iii) alkyl species 1@Al_2_O_3-700_ (c) from molecular complex 1*via* surface organometallic chemistry.

Since the discovery of Ziegler–Natta ethylene polymerization catalysts in the early 1950s,^1,2^ the nature of the active site(s) has been a matter of debate. Later, group IV transition-metal metallocenes were developed as efficient homogeneous,^3–7^ as well as supported,^8,9^ olefin polymerization catalysts for which cationic M(iv) alkyl species have been proposed as the active sites.^10^ Such species have been isolated in the form of Lewis base adducts and have been shown to be competent in olefin polymerization; one noteworthy example is Ti(iv) amidinate species [(Cp*)Ti{NC(Ph)N^i^Pr_2_}(OPPh_3_)Me][BArF_4_] (Scheme 1a).^11^ Taking into account the strong ionic character of MgCl_2_, the key support of Ziegler–Natta catalysts, surface Ti(iv) cationic alkyl species are sometimes proposed as the active sites in these systems in analogy to their metallocene equivalents.^12^

However, the evidence for Ti(iv) cationic species in Ziegler–Natta heterogeneous catalysts has remained elusive. In fact, the reaction with triethyl aluminum, a common cocatalyst used in the Ziegler–Natta process, undoubtedly leads to reduction and/or alkylation of certain sites, and Ti(iii) centers have previously been observed by XPS and EPR.^13–18^ Since their role as active sites in the polymerization of ethylene has not been evidenced so far, it remains unclear whether or not titanium d^1^ complexes can be efficient in ethylene polymerization.^19,20^ In parallel, cationic monocyclopentadienyl Ti(iii) compounds show high activities towards styrene polymerization and are catalytically comparable to or better than the corresponding Ti(iv) derivatives (Scheme 1b).^21^ Furthermore, it has been recently shown by pulsed EPR spectroscopy, combined with DFT calculations, that Ti(iii)-alkyl surface species are formed when silica-supported titanium(iii) hydride comes into contact with ethylene.^22^ This finding is consistent with previous calculations that show Ti(iii) hydrides as competent for initiating ethylene polymerization;^23^ it further implies that Ti(iii) d^1^ alkyl complexes could polymerize olefins such as ethylene.

Among well-defined Ti(iii) alkyl species, the neutral β-diiminato Ti(iii) dialkyl species (Scheme 1c, 1) are noteworthy for their stability.^24,25^ While reported to be unreactive towards ethylene polymerization without a cocatalyst at room temperature under 6 bar of ethylene,^24^ the presence of two alkyl ligands in this compound makes it particularly attractive to generate a supported Ti(iii) alkyl catalyst through surface organometallic chemistry (SOMC)^26^ and to evaluate its polymerization activity. Indeed, SOMC has been shown to provide access to many active and stable catalysts through isolation of metal sites at the surface of oxide supports.^26–29^ In addition, the presence of strong Lewis acid surface sites, such as in alumina, can also help promote the formation of more active species.^30–32^ Alternatively, cationic surface sites can be stabilized using sulfated metal oxides as a support.^32–34^

Herein, we show that the molecular β-diiminato Ti(iii) alkyl complex (Scheme 1c, 1) efficiently promotes ethylene polymerization without the need for co-catalysts at 80 °C and pressures higher than 4 bar and that its alumina-supported analogue, prepared *via* the SOMC^26^ approach (Scheme 1c, 1@Al_2_O_3-700_), shows significantly improved productivity. Using pulsed EPR spectroscopy, combined with DFT calculations and polymerization tests, we demonstrate that neutral Ti(iii) alkyl species are indeed able to initiate ethylene polymerization to produce ultra-high molecular weight polyethylene *via* ethylene insertion into a Ti(iii)–C bond. Detailed DFT calculations show that ethylene insertion into the Ti(iii)–C bond, a key step of ethylene polymerization, is favored by a partial alkylidenic character of the metal–carbon bond, as in its d^0^ analogues,^35,36^ and the added possibility of a partial electron transfer to the coordinated olefin in Ti(iii) compounds, which can be viewed as the π-back donation from the SOMO into the coordinated ethylene. This “augmented” Cossee–Arlman mechanism of olefin polymerization, possible for d^1^ metal-alkyl complexes, involves the delocalization of unpaired electrons in the transition state of olefin insertion into the metal–carbon bond with a strong alkylidenic character.

## Results and discussion

### Synthesis and characterization of neutral Ti(iii) species and examination of their polymerization activity

#### Synthesis and characterization

The molecular complex [Ti(nacnac)(CH_2_^*t*^Bu)_2_] (nacnac = [Ar]NC(Me)CHC(Me)N[Ar], Ar = 2,6-(CHMe_2_)_2_C_6_H_3_), 1 (see Scheme 1c), and its ^13^C-labelled analogue [Ti(nacnac)(^13^CH_2_^*t*^Bu)_2_], 1*, were prepared from [Ti(nacnac)Cl_2_] and LiCH_2_^*t*^Bu or its ^13^C-labeled analogue, Li^13^CH_2_^*t*^Bu, respectively, according to literature procedures.^25^ Complex 1 was characterized by NMR (see Fig. S1‡) and room-temperature CW EPR (see Fig. S2‡), consistent with its previous characterization.^25^ Complex 1 was further grafted onto alumina, partially dehydroxylated at 700 °C (Al_2_O_3-700_), resulting in material 1@Al_2_O_3-700_ (Scheme 1c). During grafting, the emerald green solution of 1 becomes colorless, while the alumina support turns grey. This reaction is accompanied by the release of 0.54 equivalents of ^*t*^BuCH_3_ per initial surface OH group (for further experimental details see ESI Part 2.1‡).

Elemental analysis of 1@Al_2_O_3-700_ gives 0.24 wt% Ti, 2.06 wt% C, 0.20 wt% N and 0.24 wt% H, corresponding to 41.0 ± 1 C/Ti (34 expected), 3.4 ± 1 N/Ti (2 expected) and 57.0 H/Ti (52 expected). The grafting of 0.54 Ti per surface OH group is thus consistent with the formation of a monografted species, [(Al_s_O)Ti(nacnac)(CH_2_^*t*^Bu)] (Al_s_ = surface aluminium). Further characterization of 1@Al_2_O_3-700_ by Fourier transform infrared spectroscopy (FTIR) shows the disappearance of the initial isolated hydroxyl groups and the appearance of a broad band from 3420 to 3760 cm^−1^, associated with hydroxyl groups interacting with the ligands of the grafted Ti centres (*e.g.* nacnac ligand).^37^ These fragments are also revealed *via ν*(C–H) vibrations at 3081–2874 cm^−1^ (Fig. S3‡).

**Fig. 1:**
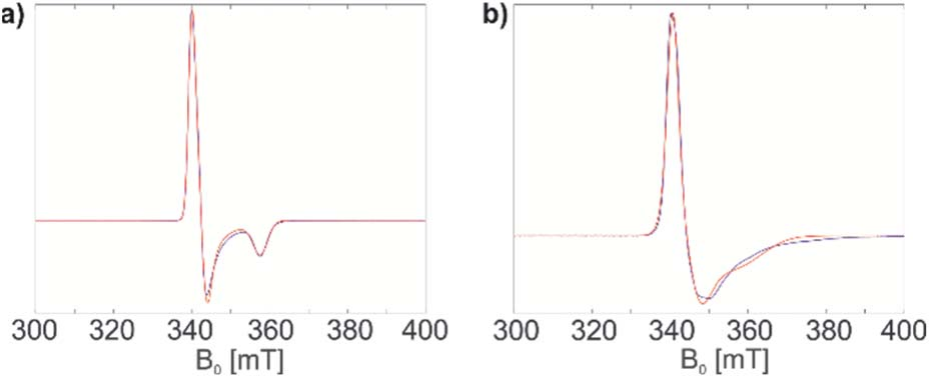
(a) CW EPR spectrum of 1 in a frozen toluene solution (blue) and simulation (red); (b) CW EPR spectrum of 1@Al_2_O_3-700_ (blue) and simulation (red). See text for simulation parameters.

Both materials 1 and 1@Al_2_O_3-700_ were then characterized with CW EPR spectroscopy. The X-band CW EPR spectrum of complex 1 recorded at 10 K (Fig. 1a, blue) originates from an *S* = 1/2 electron spin system with a nearly axial *g* tensor, having its principal values *g*_*x*_ = 1.898 ± 0.023, *g*_*y*_ = 1.981 ± 0.016, and *g*_*z*_ = 1.996 ± 0.012 (the given intervals indicate Gaussian distributions of *g* principal values; the simulation of the EPR spectrum is shown in Fig. 1a, red).

For the surface-grafted material 1@Al_2_O_3-700_, the X-band CW EPR spectrum (Fig. 1b blue) was measured at 10 K; the spectrum is consistent with the presence of an *S* = 1/2 system, with *g*_*x*_ = 1.880 ± 0.087, *g*_*y*_ = 1.970 ± 0.049 and *g*_*z*_ = 1.984 ± 0.016 (the simulation is shown in Fig. 1b, red) associated with a paramagnetic Ti(iii) surface species. Significant line broadening is observed in the grafted material 1@Al_2_O_3-700_ compared to molecular complex 1 which may result from the presence of different Ti^3+^ surface species, possibly due to small differences in local surface environments and thereby coordination geometry. In spite of replacing one Ti-coordinated carbon atom by a more electronegative oxygen atom, the *g* principal values for both 1 and 1@Al_2_O_3-700_ agree with each other within the given line widths, thus indicating a similar electronic structure and symmetry of molecular complex 1 and alumina-supported species 1@Al_2_O_3-700_.

#### Polymerization activity

**Fig. 2:**
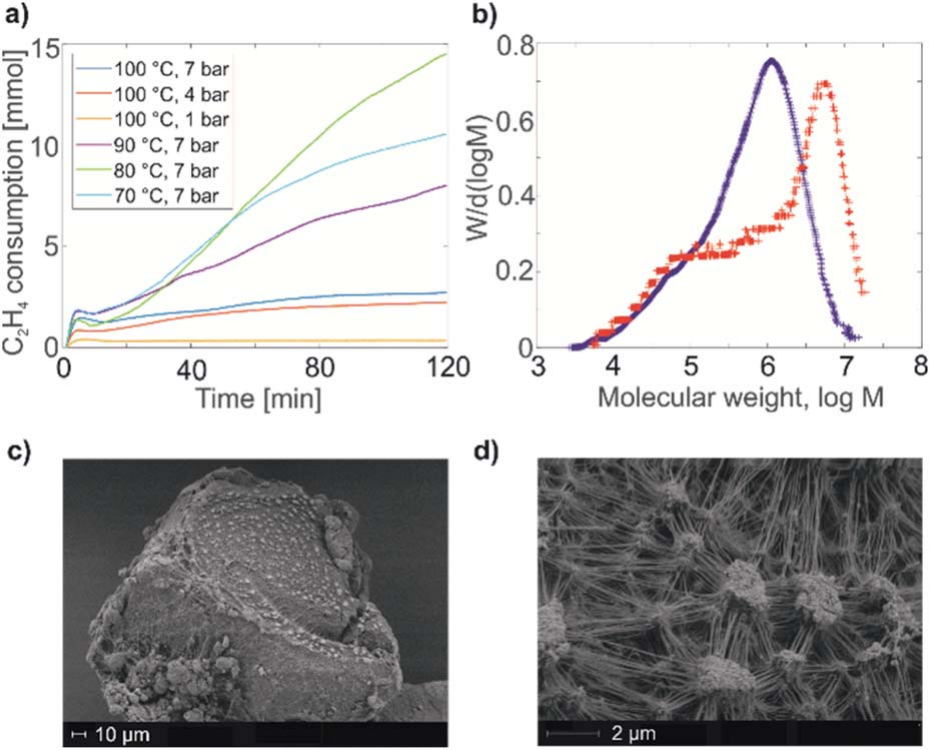
(a) Ethylene consumption of a toluene solution of 1 with time under different pressure and temperature conditions. (b) Molecular weight distribution of polyethylene produced by 1 under 7 bar of ethylene pressure and at 100 °C (blue), together with molecular weight distribution of polyethylene produced by 1@Al_2_O_3-700_ under 6 bar of ethylene pressure and at 50 °C (red). (c) SEM image of the 1@Al_2_O_3-700_ material before polymerization. (d) SEM image of the 1@Al_2_O_3-700_ material after polymerization under 1 bar of ethylene pressure and at room temperature. The changes in ethylene consumption curves up to minute 12 in (a) are due to the pressure changes in the system while reaching the reaction temperature.

We further examined the polymerization activity of 1 and its supported analogues. Molecular complex 1 was found to be active in ethylene polymerization in the temperature range 80–100 °C and at ethylene pressures higher than 4 bar (Fig. 2a). The reaction was carried out in either benzene or toluene solutions. The formation of white films of polyethylene (PE) was observed after 2 hours. Within the range tested, a maximal calculated productivity of 11 kg_PE_ (mol_Ti_ h)^−1^ was achieved at 80 °C under 7 bars of ethylene in toluene solution (Fig. 2a, green). Note that heating a toluene solution of 1 under the same conditions (80 °C), but in the absence of ethylene, showed neither a white film nor the formation of any new species visible by EPR or NMR spectroscopies (Fig. S4‡). We can therefore propose that 1 is a direct precursor of the active species that are formed under ethylene pressure.

The material 1@Al_2_O_3-700_ is significantly more active towards ethylene polymerization than its molecular analogue 1, and the polymerization stops within minutes due to the formation of a dense PE layer that can be directly observed with scanning electron microscopy (SEM) (Fig. 2c and d). Overall, this material displays a productivity of *ca.* 36 kg_PE_ (mol_Ti_ h)^−1^ under 6 bar of ethylene at 50 °C. Note that, in contrast to 1, 1@Al_2_O_3-700_ initiates ethylene polymerization even under very mild conditions, *i.e.* room temperature, 80 mbar of ethylene.

The molecular weight distribution for PE, produced by 1 and 1@Al_2_O_3-700_, is obtained with size-exclusion chromatography (SEC, Fig. 2b). For both catalysts, the distribution is asymmetric with a main heavy fraction and a broad distribution of molecular weights of lighter PE fractions, possibly being the products of chain termination reactions. The main fraction of PE has a molecular weight of *ca.* 1130 kg mol^−1^ for 1 and *ca.* 5660 kg mol^−1^ for 1@Al_2_O_3-700_, which is typical for ultra-high molecular weight polyethylene (UHMWPE).^38^ As the PE molecular weight distribution has similar character for both 1 and 1@Al_2_O_3-700_, and as UHMWPE is produced in both cases, we propose that the polymerization on both catalysts takes place *via* a similar mechanism. The higher molecular weight of the main fraction of PE, produced with 1@Al_2_O_3-700_, can be explained by the absence and/or the slower rate of possible chain termination reactions, one example being the α-H abstraction reaction.

#### Detailed EPR characterization and evaluation of the structures of 1 and 1@Al_2_O_3-700_

**Fig. 3:**
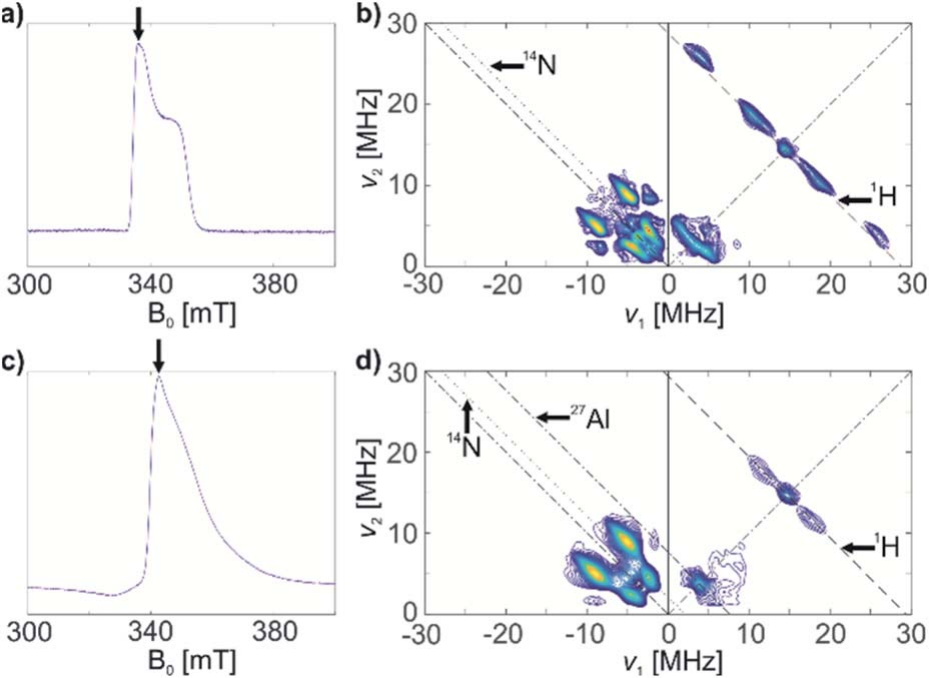
(a) Echo-detected EPR spectrum of a frozen toluene solution of 1; (b) HYSCORE spectrum of 1, *τ* = 128 ns; (c) echo-detected EPR spectrum of 1@Al_2_O_3-700_; (d) HYSCORE spectrum of solid 1@Al_2_O_3-700_, *τ* = 128 ns. Arrows on the echo-detected EPR spectra (a and c) indicate the magnetic field positions for HYSCORE measurements. Antidiagonal lines on the HYSCORE spectra (b and d) correspond to nuclear frequencies indicated on the spectra (black arrows).

We further characterize complex 1 and the associated surface species in 1@Al_2_O_3-700_ by pulse EPR spectroscopy, namely by HYSCORE (Hyperfine Sublevel Correlation Spectroscopy).^39^ This method was selected for its ability to observe weak hyperfine couplings (*e.g.* weakly coupled ^14^N and ^1^H) that are usually not resolved in the CW EPR spectra. The X-band HYSCORE spectra of 1 and 1@Al_2_O_3-700_, shown in Fig. 3b and d, respectively, were measured at 10 K at the field positions corresponding to the maxima of the echo-detected EPR spectra (Fig. 3a and c; the field positions are marked with arrows). Both spectra shown in Fig. 3b and d were measured with an interpulse delay of *τ* = 128 ns. The X-band HYSCORE spectra were also measured with three *τ* values *τ* = 128 ns, 160 ns and 224 ns to avoid loss of spectral information due to blind spots (see Fig. S5‡ for the *τ*-summation spectra); however, it appeared that the spectra with *τ* = 128 ns contained all peak patterns present in the *τ*-summation spectra except for the ^1^H matrix peak, whose suppression is favorable.

The HYSCORE spectrum of complex 1 (Fig. 3b) shows the presence of ^1^H and ^14^N nuclei in the Ti(iii) coordination sphere, revealed by cross peaks along the ^1^H antidiagonal in the weak coupling (+, +) quadrant, corresponding to ^1^H hyperfine couplings, and by peaks in the low-frequency region both in the weak coupling (+, +) and strong coupling (−, +) quadrants, corresponding to ^14^N hyperfine and quadrupole couplings. For the material 1@Al_2_O_3-700_, the HYSCORE spectrum (Fig. 3d) reveals the presence of ^14^N hyperfine and quadrupole couplings as well, which are close to the ones observed for molecular complex 1 before grafting. This indicates that the (nacnac) ligand remains coordinated to the Ti(iii) center after grafting onto the Al_2_O_3-700_ surface. For 1@Al_2_O_3-700_, a different set of ^1^H hyperfine couplings is observed, with a loss of strongly coupled ^1^H being the most prominent change after grafting. Most probably, this is due to the loss of one of the CH_2_^*t*^Bu ligands upon grafting, as shown in Scheme 1. Furthermore, ^27^Al couplings are observed for 1@Al_2_O_3-700_ as a matrix peak on the ^27^Al antidiagonal line (Fig. 3d) and are well-resolved in Q-band HYSCORE spectra (Fig. 5c). This is consistent with the presence of nearby surface Al atoms in the surroundings of Ti(iii), as expected for a grafted species.

Using the experimental HYSCORE spectra, we could further estimate the conformation of molecular complex 1 in toluene solution and provide detailed structural information regarding the surface species in 1@Al_2_O_3-700_ by comparing experimental and calculated hyperfine and quadrupole tensors. In order to find the explicit structure of complexes 1 and 1@Al_2_O_3-700_, the X-ray crystal structures of 1 (ref. 25) (see Fig. S6‡) and the derived model for the species in 1@Al_2_O_3-700_ were optimized with unrestricted Kohn–Sham density functional theory (DFT), using the functional PBE0 (ref. 40) in ORCA 3.^41,42^ For these geometry optimizations, a polarized triple-ζ def2-TZVPP basis set^43^ was used for all atoms, together with Becke's three-center dispersion correction.^44^ The COSMO continuum solvation model^45^ was applied for complex 1. Furthermore, the hyperfine and quadrupole tensor parameters were calculated with the def2-TZVPP basis set for Ti and Al atoms and the EPR-II basis set^46^ for all other atoms. Based on the calculated parameters, the simulations of HYSCORE spectra were carried out in EasySpin.^47^ Hyperfine and nuclear quadrupole couplings were previously found to be highly sensitive to small structural changes.^22^ Here we find that rotation of one of the CH_2_^*t*^Bu ligands of 1 has a tremendous effect on the calculated isotropic part of the ^1^H hyperfine tensors of α-H atoms of the CH_2_^*t*^Bu ligands (see Fig. S7‡). Together with their dipolar part, which is sensitive to the Ti–H distances, the calculated hyperfine tensors act as fingerprints of α-H positions in the calculated structures of 1 and 1@Al_2_O_3-700_. The calculated multiple-quantum (*e.g.* double-quantum) ^14^N transitions are affected by such a rotation as well (see Fig. S7‡), and hence are an effective probe for the molecular structure and conformation through both hyperfine and quadrupole tensors. This allows us to use DFT-based simulations of the experimental HYSCORE spectra as a tool for evaluation of the molecular structures of 1 and 1@Al_2_O_3-700_. After such an evaluation, the explicit structures and the Kohn–Sham molecular orbital sets for 1 and 1@Al_2_O_3-700_ were obtained simultaneously, both verified by a comparison of the experimental and simulated HYSCORE spectra.

**Fig. 4:**
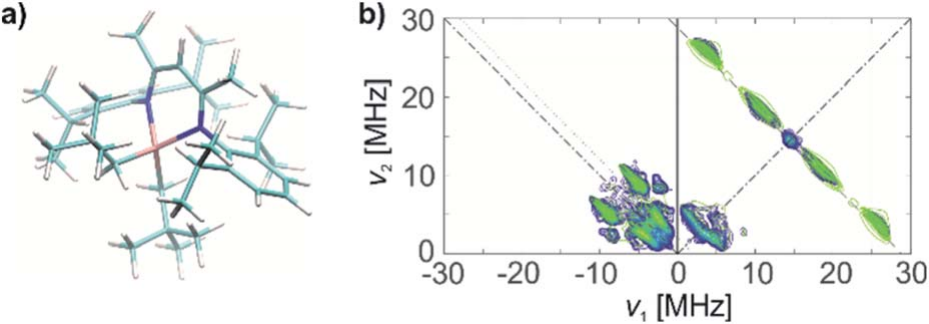
(a) Molecular structure of 1, based on DFT optimizations and on the agreement with HYSCORE spectra. (b) X band HYSCORE spectrum of 1 (blue) and simulation (green), based on DFT calculations on the structure in (a).

The crystal structure of 1, however, does not yield the correct set of ^1^H hyperfine coupling parameters, since it fails to fully predict the experimental HYSCORE spectrum (Fig. S6‡). This indicates that complex 1 possesses a different conformation in frozen toluene solution than in the solid state. However, after the geometry optimization using the parameters indicated above, the obtained conformation (Fig. 4a) generates hyperfine and quadrupole tensors for ^14^N and ^1^H nuclei (Table 1), which simulate the entire X-band HYSCORE spectrum rather nicely (Fig. 4b). Both the isotropic and dipolar parts of ^1^H hyperfine tensors fit well to the experimental spectrum in Fig. 4b, thus indicating the correct positions of the α-H atoms of CH_2_^*t*^Bu ligands in the optimized structure (Fig. 4a). Furthermore, the calculated ^14^N hyperfine and quadrupole tensor parameters are found to simulate both the X-band (Fig. 4b) and Q-band (see Fig. S8‡) HYSCORE spectra. We therefore propose that in a frozen toluene solution, molecular compound 1 is present in the form of the conformer shown in Fig. 4a, which differs from the solid-state structure by a slight rotation of the CH_2_^*t*^Bu ligands around the Ti–C axis (see ESI Part 2.3‡).

**Table 1:** Calculated hyperfine and quadrupole couplings for EPR active nuclei (in MHz) for the structures of 1 and 1@Al_2_O_3-700_, together with ^27^Al hyperfine couplings for 1@Al_2_O_3-700_, based on least squares fitting of the experimental Q-band HYSCORE spectrum

	1	1@Al_2_O_3-700_
^1^H	*a*_iso_ = 7.77	*a*_iso_ = 5.80
	*a*_dip_ = [−4.94; −3.08; 8.02]	*a*_dip_ = [−3.87; −2.57; 6.44]
^1^H	*a*_iso_ = 13.37	*a*_iso_ = −3.34
	*a*_dip_ = [−3.85; −2.57; 6.42]	*a*_dip_ = [7.65; −3.34; −4.30]
^1^H	*a*_iso_ = 18.76	—
	*a*_dip_ = [−3.93; −2.82; 6.76]	
^1^H	*a*_iso_ = 1.27	—
	*a*_dip_ = [−3.40; −5.71; 9.11]	
^14^N	*a*_iso_ = −7.98	*a*_iso_ = −6.37
	*a*_dip_ = [2.05; 1.74; −3.79]	*a*_dip_ = [0.61; −0.17; −0.43]
	*P* = 2.72 (*η* = 0.316)	*P* = 2.50 (*η* = 0.470)
^14^N	*a*_iso_ = −7.07	*a*_iso_ = −7.03
	*a*_dip_ = [1.08; 0.74; −1.82]	*a*_dip_ = [0.80; 0.50; −1.30]
	*P* = 2.69 (*η* = 0.276)	*P* = 2.60 (*η* = 0.354)
^27^Al (least squares fit)	—	*a*_iso_ = 2.68
		*a*_dip_ = [−2.23; 3.56; −1.34]
		*P* = 7.66 (*η* = 0.01)

A similar approach of evaluating the molecular structure was used for the grafted species 1@Al_2_O_3-700_. We considered two possible types of 1@Al_2_O_3-700_ species, either neutral [(Al_s_O)Ti(nacnac)(CH_2_^*t*^Bu)] surface Ti complexes obtained *via* grafting of 1 through surface OH groups (Scheme 1c) or cationic [Ti(nacnac)(OAl_s_)]^+^⋯[(CH_2_^*t*^Bu)Al_s_]^−^ where the alkyl group is transferred onto alumina (see Fig. S9‡) as previously observed for other complexes.^31,48,49^ Geometry optimizations with subsequent calculations of EPR parameters were performed for both cationic and neutral models. It appeared that the cationic model, however, does not result in ^1^H hyperfine couplings that are strong enough to simulate the elongated experimental HYSCORE ridges (see Fig. S9‡). The α-H atoms of the CH_2_^*t*^Bu fragment in this model are far from the Ti center such that both dipolar and isotropic parts of the ^1^H hyperfine tensors appear too small (see ESI Part 2.3‡). Only the neutral models exhibit ^1^H hyperfine couplings that are strong enough to simulate the experimental HYSCORE spectrum.

The geometry optimization of the neutral [(Al_s_O)Ti(nacnac)(CH_2_^*t*^Bu)] model was performed as described above for complex 1, followed by a DFT-based calculation of EPR parameters. The complex nature of the Al_2_O_3_ surface results in a number of different types of OH groups that can participate in grafting.^31^ Here, we represent the (OH)Al_s_ surface atom by the simplest possible tetracoordinated neutral Al model, namely (HO)Al_s_ = (HO)Al(OH)_2_(H_2_O). Within this approximation, the symmetry of the {AlO_4_} cluster may be decreased compared to the structure of an alumina surface center due to different Al–OH_2_ and Al–OH bond lengths; this may result in overestimation of quadrupole coupling for the ^27^Al nucleus. Furthermore, it is worth noting that the spin density on Al nuclei tends to be underestimated even with basis sets that contain diffuse functions, which are expected to better describe spin density near the nucleus.^50^ Taking this into account, it appeared to be better to rely on experimentally determined ^27^Al hyperfine and quadrupole couplings rather than to evaluate these from DFT calculations.

**Fig. 5:**
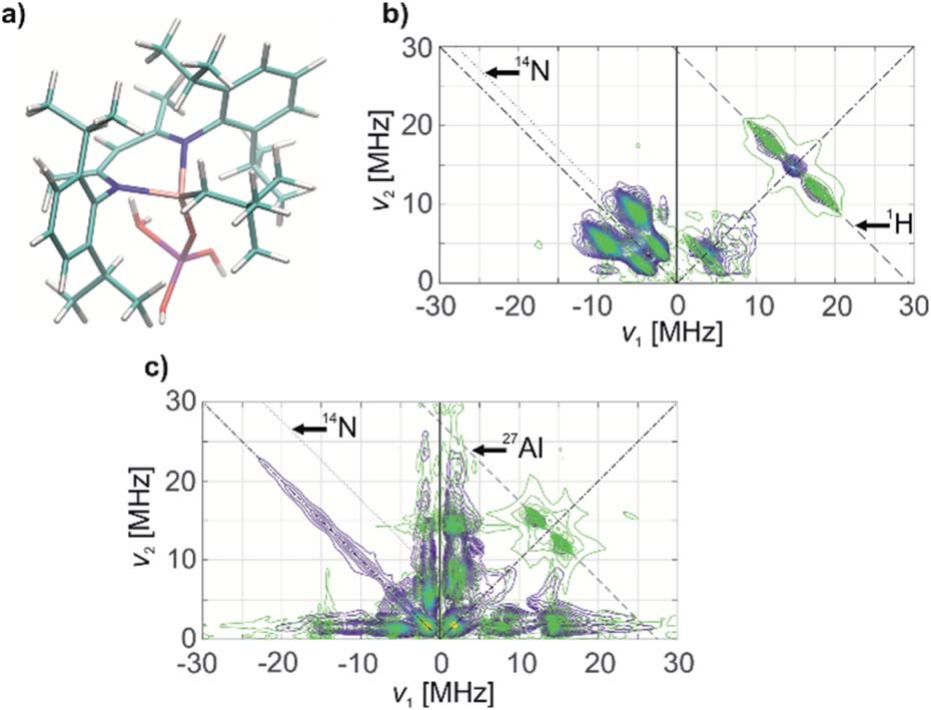
(a) Model for 1@Al_2_O_3-700_ species representing a neutral [(Al_s_O)Ti(nacnac)(CH_2_^*t*^Bu)] complex based on agreement with HYSCORE spectra. (b) X-band HYSCORE spectrum of 1@Al_2_O_3-700_ (blue) and simulation (green), based on DFT calculations on the obtained model. (c) Q-band HYSCORE spectrum of 1@Al_2_O_3-700_ (blue) and simulation (green), based on DFT computed EPR parameters from the obtained model (a) for ^14^N, combined with EPR parameters for ^27^Al, obtained from a least squares fit of the experimental spectrum. The ridge on the anti-diagonal in the left quadrant is due to an echo crossing that was not fully suppressed by phase cycling.

The ^1^H and ^14^N couplings (Table 1) computed for the thus-obtained model (Fig. 5a) simulate the experimental X-band HYSCORE spectrum quite well (Fig. 5b). Similar to molecular complex 1, this indicates the correct positions of α-H atoms of a CH_2_^*t*^Bu ligand in the optimized structure. Although ^27^Al couplings are not detected in the X-band HYSCORE spectrum, Q-band HYSCORE (Fig. 5c) provides the necessary information to determine the ^27^Al hyperfine couplings by least squares fitting (Fig. S10‡). Together with the calculated ^14^N hyperfine couplings, they fit reasonably to the Q-band HYSCORE spectrum (Fig. 5c, green). This allows us to consider the obtained model (Fig. 5a) as an explicit structure of Ti(iii) surface species observed in our EPR studies. Therefore, we confirm that grafting of 1 onto the Al_2_O_3-700_ surface yields a neutral Ti(iii) alkyl species, namely [(Al_s_O)Ti(nacnac)(CH_2_^*t*^Bu)], with the structure shown in Fig. 5a. The production of UHMWPE by 1@Al_2_O_3-700_ as well as by 1, together with a high polymerization activity of 1@Al_2_O_3-700_, is consistent with a predominant presence of Ti(iii) neutral alkyl species on the Al_2_O_3-700_ surface after grafting.

#### ^13^C labelling as a tool to probe Ti-alkyl chains and the polymerization mechanism

In order to gain further insight into the polymerization mechanism and the active state of the catalyst, we performed EPR studies on the molecular complex [Ti(nacnac)(CH_2_^*t*^Bu)_2_] after reaction with C_2_H_4_, in combination with ^13^C isotope labelling. Among two possible labelling schemes (Scheme S1‡), we decided to use the one that involves the reaction of non-labelled ethylene with selectively ^13^C-labelled complex [Ti(nacnac)(^13^CH_2_^*t*^Bu)_2_] (1*) and that should yield (Ti^III^–(CH_2_CH_2_)_*n*−1_(^13^CH_2_^*t*^Bu)) with a labelled ^13^CH_2_^*t*^Bu terminating group. This reaction should lead to reduction of the initial ^13^C signal intensity in the EPR spectra of 1*, which may be observed with pulse hyperfine EPR methods and interpreted by comparison of the spectra before and after the reaction. Using the alternative labelling scheme, which involves the reaction of ^13^C labelled ethylene with the unlabelled complex [Ti(nacnac)(CH_2_^*t*^Bu)_2_] (1), proved to be difficult and did not allow the detection of ^13^C hyperfine couplings (see the ESI‡ for details), possibly due to a broad distribution of conformations associated with the flexibility of PE ligands ((^13^CH_2_)_*n*_(CH_2_^*t*^Bu)). Such distribution would result in a broad set of ^13^C hyperfine couplings and thereby broad spectral lines unobservable in our hyperfine EPR experiments.

**Fig. 6:**
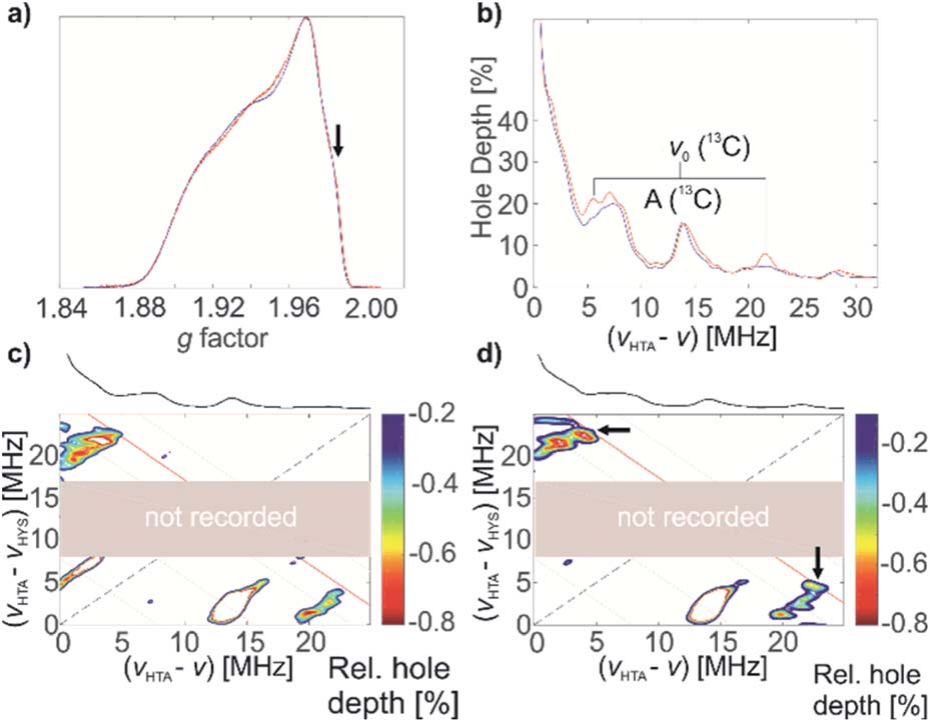
(a) Q-band echo-detected EPR spectra of 1 (blue) and 1* (red), intensities normalized; the position of further measurements is indicated with an arrow. (b) 1D CHEESY-detected NMR spectra of 1 (blue) and 1* (red); the ^13^C signals are marked on the spectrum. (c) Q-band 2D CHEESY-detected NMR spectrum of 1; the ^13^C nuclear Zeeman frequency is shown by a red antidiagonal line. (d) Q-band 2D CHEESY-detected NMR spectrum of 1*; the ^13^C nuclear Zeeman frequency is shown by a red antidiagonal line along which cross peaks due to ^13^C hyperfine coupling are observed (marked with arrows). The gray area has not been recorded during the experiment in order to optimize sensitivity and the spectra were recorded at slightly different fields and frequencies.

^13^C-labeled complex 1* was characterized by pulse EPR spectroscopy. It exhibits the same echo-detected EPR spectrum and, consequently, the same *g* tensor parameters as non-labelled complex 1 (Fig. 6a). Detection of the ^13^C couplings of the coordinating (^13^CH_2_^*t*^Bu) ligands proved difficult. According to DFT calculations for the optimized structure of 1 (Fig. 4a), the ^13^C hyperfine tensors for both α-C atoms of (^13^CH_2_^*t*^Bu) ligands of complex 1* show large couplings that are mostly isotropic (*a*_iso_ = −20.51 MHz, *a*_dip_ = [1.26 0.54 –1.80] MHz and *a*_iso_ = −21.69 MHz, *a*_dip_ = [1.52 0.50 –2.01] MHz for the two ^13^C nuclei). This leads to a low probability of the forbidden electron-^13^C-nuclear spin transitions, making direct observation of the ^13^C signals with ESEEM-based techniques (*e.g.* HYSCORE) difficult (in the Q band) or impossible (in the X band). Furthermore, strong ^14^N ESEEM modulations in both the X- and Q-band may suppress the ^13^C modulations due to a cross-suppression effect.^51^ For this reason, we used an alternative EPR methodology based on the recently developed hyperfine technique CHEESY-detected NMR (CHEESY = chirp echo EPR spectroscopy).^52^ This method is based on long selective hole burning pulses that drive forbidden transitions, similar to ELDOR-detected NMR, but the detection is based on broadband chirp echoes and subsequent Fourier transform. This leads to a multiplex advantage and, consequently, to higher sensitivity (see ESI Part 2.4‡ for more details). Indeed, the ^13^C signals were observed already in the 1D CHEESY-detected NMR spectrum of 1*, revealed in comparison with the same spectrum of non-labelled complex 1 where the ^13^C signals were not observed (Fig. 6b).

These ^13^C signals, observed at the orientation corresponding to *g* = 1.983 (Fig. 6a, marked with an arrow), are better resolved in two-dimensional HYSCORE-type CHEESY-detected NMR spectra (Fig. 6c and d), which are obtained by applying a selective π pulse with variable frequency before the HTA pulse (see ESI Part 2.4‡ for the details of CHEESY-detected NMR experiments). The comparison of the spectra for 1 (Fig. 6c) and 1* (Fig. 6d) reveals the peaks at (21, 5) MHz, corresponding to the signals of ^13^C in [Ti(nacnac)(^13^CH_2_^*t*^Bu)_2_]. Based on the obtained spectra (Fig. 6b–d), the ^13^C hyperfine coupling was estimated to be *a*_iso_(^13^C) = 16 MHz. This allowed us to confirm the assignment by Q-band HYSCORE and Q-band Davies ENDOR^53^ (see Fig. S11‡), where the weak spectral signals corresponding to ^13^C hyperfine couplings were identified by comparison to the 1D- and 2D-CHEESY detected NMR spectra.

**Fig. 7:**
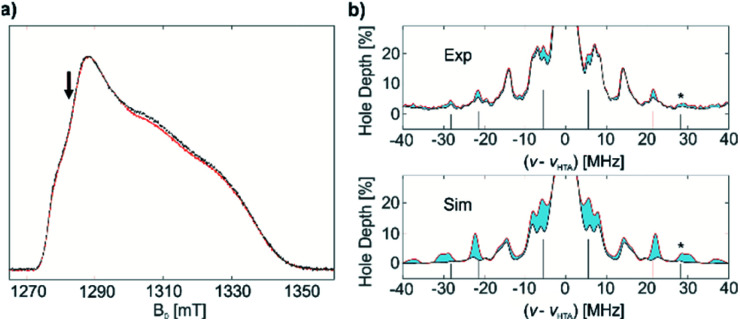
(a) Q-band echo-detected EPR spectra of 1* (red) and 1* + C_2_H_4_ (black), intensities normalized; the position of further measurements is indicated by an arrow. (b) Top: experimental 1D CHEESY-detected NMR spectra of 1* (red) and 1* + C_2_H_4_ (black); no normalization was applied. Bottom: simulated 1D CHEESY-detected NMR spectra of 1* (red) and 1 (black). ^13^C signals, corresponding to *A*(^13^C) = 16 MHz are marked with lines under the spectra; the combination signal of (^13^C + ^14^N) is marked with an asterisk. The difference between the spectra due to difference in ^13^C signal intensities is colored in blue.

Next, a benzene solution of 1* was brought into contact with C_2_H_4_ (1000 equivalents) at 80 °C for 2 hours. After the reaction, the excess C_2_H_4_ was removed and EPR measurements of (1* + C_2_H_4_) were performed. The similarities in the echo-detected Q-band EPR spectra (Fig. 7a) of complex 1* before and after polymerization indicate similar *g* tensor parameters consistent with conservation of the symmetry of Ti(iii). Using the same methodology as before, 1D CHEESY-detected NMR spectra before and after the polymerization were measured (Fig. 7b) at the same frequency and field positions, with an identical microwave resonator profile (Fig. S12‡). Although the absolute echo intensities for both samples may still be slightly different, the CHEESY-detected NMR signals, being essentially the ratio of the spectra with and without a high turning-angle pulse, can be considered a quantitative tool to probe the amount of EPR active nuclei in the Ti(iii) coordination sphere before and after the reaction with C_2_H_4_.

An obvious decrease of the ^13^C signal intensity is observed after polymerization (Fig. 7b, top) for all previously observed ^13^C lines of 1*, corresponding to *a*_iso_(^13^C) = 16 MHz, as well as for the combination signal of (^13^C + ^14^N). At the same time, the spectral lines, determined by ^14^N hyperfine and quadrupole couplings (*e.g.* double-quantum ^14^N signals around 14 MHz), are the same before and after polymerization both regarding their frequencies and intensities. Since the frequencies of these ^14^N signals are sensitive even to small changes in the structure and conformation of 1* (see Fig. S7‡) we can conclude that the structure of the Ti(iii) coordination sphere experiences minimal change upon polymerization. These ^14^N signals were reasonably simulated with the calculated values for the previously estimated conformation of 1 (Fig. 4a) in the spectra both before and after polymerization of ethylene (Fig. 7b, bottom). Together with the observed decrease of the ^13^C signal intensity, this indicates that ligand exchange of ^13^CH_2_^*t*^Bu to (CH_2_CH_2_)_*n*−1_(^13^CH_2_^*t*^Bu) occurs with preservation of the initial structure and conformation of 1*. Indeed, the experimentally observed decrease of ^13^C signals is simulated well as a difference between 1D CHEESY-detected NMR simulations for labelled complex 1* and non-labelled complex 1 (Fig. 7b, bottom). To that end, the experimental *a*_iso_(^13^C) = −16 MHz, together with DFT computed *a*_dip_(^13^C) parameters, was used for the simulation of the spectrum of 1* (see ESI Part 2.4‡ for the details of the simulation). The comparison of the simulated and experimentally observed decrease of the ^13^C signal intensity implies that probably not all the complex 1* present is affected by the ligand exchange, but only a part of it. This indicates that only a part of molecules of 1* acts as active centres of ethylene polymerization under the aforementioned reaction conditions. This is consistent with the presence of an induction period at the beginning of polymerization, revealed by changes in ethylene consumption (Fig. 2a). It is also consistent with the calculated energy barrier for the first olefin insertion (*vide infra*). An exact quantification is difficult without precise knowledge of the full ^13^C hyperfine tensor, which also affects line intensities.

Based on the discussed experimental results, we propose that olefin polymerization takes place *via* C_2_H_4_ insertion into the Ti(iii)–C bond in the molecular system Ti(nacnac)(CH_2_R)_2_–1. Unfortunately, we were not able to study ethylene polymerization with 1@Al_2_O_3-700_ due to *T*_2_ electron spin relaxation times, which are *ca.* 6 times shorter for 1@Al_2_O_3-700_ than for 1. This limits the observation window length and, consequently, the resolution of CHEESY-detected NMR^26^ such that the separation of ^13^C, ^14^N and ^27^Al signals becomes rather uncertain. However, the molecular weight distributions of produced PE are quite close for both 1 and 1@Al_2_O_3-700_, with UHMWPE being produced in both cases. This indicates a similar type of active center and polymerization mechanism for both catalysts. As we determined a neutral [(Al_s_O)Ti(nacnac)(CH_2_^*t*^Bu)] species to be present in 1@Al_2_O_3-700_, which is similar in terms of structure and electronic properties to molecular complex 1, we propose that ethylene polymerization on 1@Al_2_O_3-700_ proceeds *via* the same mechanism as for 1, *i.e.* through C_2_H_4_ insertion into the Ti(iii)–C bond in [(Al_s_O)Ti(nacnac)(CH_2_^*t*^Bu)].

### Electronic structures and the polymerization mechanism for 1 and 1@Al_2_O_3-700_

#### α-Agostic C–H interaction and π character of Ti–C bonds of 1 and 1@Al_2_O_3-700_

The estimated structure of molecular complex 1 (Fig. 4a) is notable because of the presence of unusually small Ti–C–H angles in both Ti alkyls. These angles of 95.56° and 96.95° are associated with relatively short Ti⋯H distances (2.500 Å and 2.503 Å, respectively) as well as with elongated C_α_–H distances (up to 1.104 Å and 1.107 Å, respectively), in comparison with typical distances of 1.091–1.103 Å for all the other aliphatic C–H distances in 1. Such observations are consistent with the presence of so-called α-agostic C–H bonds in this Ti(iii) compound. A similar, but weaker, α-H agostic Ti⋯H interaction is found for the refined structure of alumina-grafted neutral species [(Al_s_O)Ti(nacnac)(CH_2_^*t*^Bu)] of 1@Al_2_O_3-700_ (further named 1@Al_2_O_3-700_), with one of the α-H atoms of the CH_2_^*t*^Bu ligand having a Ti–C–H angle of 101.80° and a Ti–H distance of 2.603 Å.

Essentially, α-agostic C–H bonds are described^35,54^ as the donation of electrons from the filled molecular orbital corresponding to the C–H bond to a metal d-orbital of appropriate symmetry that is empty for d^0^ metals. This agostic interaction has been recently related to a metal–carbon bond acquiring a π (or alkylidene) character,^35,55^ which favors the olefin insertion process. The degree of this π character could be indirectly estimated from the deviation of the Ti–C–H angle from 109° towards *ca.* 90°. In order to estimate directly the π character of Ti(iii)–C bonds of 1 and 1@Al_2_O_3-700_, a Natural Bond Orbital (NBO) analysis^56^ was performed, using the program NBO 7.0.^57^ The molecular orbital sets for NBO analyses were generated using ORCA 4 (ref. 42) with the same parameters for the DFT calculations as the ones used for the simulations of the HYSCORE spectra (PBE0 functional together with the def2-TZVPP basis set for Ti and Al atoms and EPR-II basis set for all other atoms). Given the good agreement between the measured and calculated HYSCORE spectra, this computational method describes the electronic structures of 1 and 1@Al_2_O_3-700_ with sufficient accuracy.

**Fig. 8:**
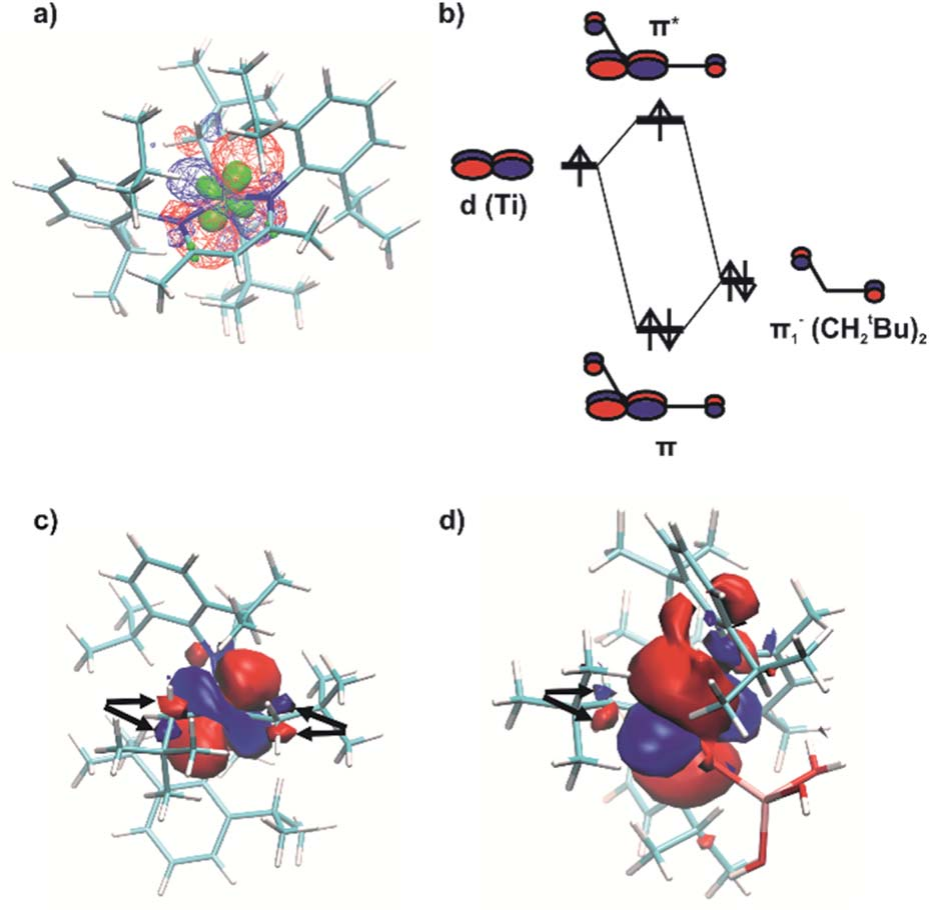
(a) A natural spin-α orbital, related to the SOMO of 1 (red for positive and blue for negative signs of the wavefunction, the same in all the panels), together with the spin density distribution (green). (b) Qualitative molecular orbital diagram, illustrating the formation of the π*(Ti–(CH_2_^*t*^Bu)_2_) orbital being the SOMO component of 1. (c) Natural SOMO of 1 as visible from the (CH_2_^*t*^Bu)_2_ side, showing a central d-type part and two carbon p-type parts (marked with arrows). (d) Natural SOMO of 1@Al_2_O_3-700_, showing a carbon p-type part (marked with arrows).

The NBO analysis revealed a natural orbital, related to a singly occupied molecular orbital (SOMO) of paramagnetic complex 1 (Fig. 8a, red and blue). Its shape correlates well with the calculated distribution of spin density in space (Fig. 8a, green), thus confirming the close relation of this natural orbital to the SOMO. This orbital is nearly axially symmetric, which is consistent with the experimentally observed axial symmetry of the *g* tensor.

The spatial distribution of the SOMO-related natural orbital of 1 includes four lobes of the p-type on two nitrogen atoms and two carbon atoms of CH_2_^*t*^Bu ligands, all being in antiphase with the central d-type lobes (Fig. 8a and c). The part of this orbital, which includes the central d-type part and the two carbon p-type parts (Fig. 8c), can be understood as a product of interaction of the half-filled d_*xy*_-type Ti orbital and one of two degenerate filled π^−^ orbitals of the (CH_2_^*t*^Bu)_2_ fragment (Fig. 8b). This orbital features π* symmetry with respect to the Ti–C bond.

Therefore, the presence of the π* orbital, as part of the SOMO and delocalized between two CH_2_^*t*^Bu ligands, reveals the existence of π bonding in Ti–(CH_2_^*t*^Bu)_2_. This π interaction, although being weakened by the unpaired electron in the antibonding π* orbital (Fig. 8b), stabilizes the structure with the α-agostic C–H bonds for 1. Compared to metal d^0^ complexes^35^ this α-H agostic interaction involves a half-filled metal d orbital instead of an empty one, as revealed by the NBO analysis for the refined structure of 1. Such an interaction brings a π character into both Ti–C bonds of 1. This π character is also evidenced by the deviation of the natural hybrid orbital (NHO) on carbon from the Ti–C axis (*θ*_NHO–C–Ti_ = 15.0° and 14.4° for the two Ti–C bonds of 1) – for a pure σ-bond no deviation would be expected (0.0°).^35^

A natural orbital of similar type, including a p-type lobe on the carbon atom of the single CH_2_^*t*^Bu ligand, is also found for 1@Al_2_O_3-700_ (Fig. 8d). This indicates the presence of a π* orbital and, consequently, a π interaction in the [Ti–(CH_2_^*t*^Bu)] system. However, the π character acquired by the Ti–C bond of 1@Al_2_O_3-700_ is less pronounced compared to that of complex 1 (*θ*_NHO–C–Ti_ = 9.2° for 1@Al_2_O_3-700_). The described π interaction, which involves half-filled metal d orbitals, might be present for all paramagnetic transition metal alkyl complexes, provided that the corresponding half-filled d orbitals have appropriate symmetry.

#### Olefin polymerization pathways of 1 and 1@Al_2_O_3-700_

The presence of π character in the metal–carbon bonds has been found to play a crucial role in the reactivity of d^0^ compounds, making them reactive towards olefin insertion.^35^ It was also used as an explanation for C–H activation pathways, including α-H abstraction in dialkyl compounds, that have been shown to be isolobal reactions.^58^ Indeed, the α-H abstraction is a known synthetic pathway of Ti(iv) d^0^ alkylidenes, prepared *via* oxidation of 1 by AgOTf.^25^ However, the transition state (TS) energy for the α-H abstraction process for d^1^ complex 1, calculated in ORCA 3 (ref. 41) with the same DFT parameters as the ones used for ground state optimizations, was found to be relatively high (Δ*H*^‡^_298_ = 31.5 kcal mol^−1^; Δ*G*^‡^_298_ = 31.5 kcal mol^−1^, Fig. S13‡). This should make the process slow and indicate the relative stability of 1 even under the elevated temperatures used in ethylene polymerization. In contrast, the transition state for C_2_H_4_ insertion into the Ti(iii)–C bond of 1 (Fig. S13‡) appeared to have an overall energy barrier with respect to the initial reagents (1 + C_2_H_4_) of Δ*H*^‡^_298_ = 22.5 kcal mol^−1^ and Δ*G*^‡^_298_ = 33.7 kcal mol^−1^. The large difference of 9.0 kcal mol^−1^ in the TS enthalpies (Δ*H*^‡^_298_) suggests that the reaction of C_2_H_4_ insertion into the Ti(iii)–C bond is more facile than α-H abstraction. Looking at the free energy, where entropy factors in solution are typically overestimated,^59^ one would expect that both processes can be competitive. Overall, the calculated Δ*H*^‡^_298_ and Δ*G*^‡^_298_ values of the ethylene insertion for complex 1 are consistent with a slow polymerization reaction at 80 °C as well as the need to use high pressure to conduct the reaction. In fact, similar calculated energetics are reported for the Ti(iv) homogeneous catalysts of ethylene polymerization (*e.g.* Δ*H*^‡^_298_ and Δ*G*^‡^_298_ = 16 and 28 kcal mol^−1^, respectively, for the [H_2_Si(C_5_H_4_)(^*t*^BuN)]TiCH_3_^+^⋯H_3_CB(C_6_F_5_)_3_^−^ ion pair^60^). This supports our experimental evidence of ethylene insertion into the Ti(iii)–C bond as the mechanism of ethylene polymerization of molecular catalyst 1.

**Fig. 9:**
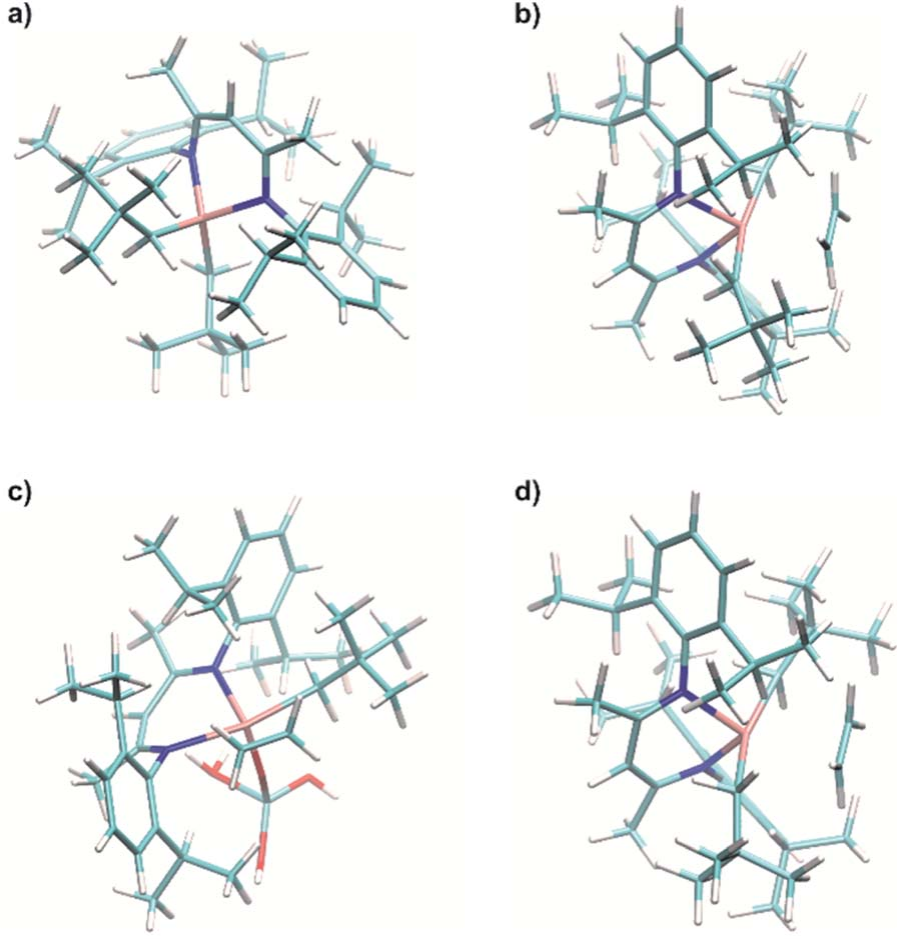
(a) Calculated structure of the model Ti(iv) complex [Ti(nacnac)(CH_2_^*t*^Bu)_2_]^+^ (1^+^). (b) Calculated structure of π-ethylene complex 1⋯C_2_H_4_. (c) Calculated structure of π-ethylene complex 1@Al_2_O_3-700_⋯C_2_H_4_. (d) Calculated structure of model Ti(iv) π-ethylene complex 1^+^⋯C_2_H_4_.

It is noteworthy that for the model Ti(iv) cationic analogue of complex 1, namely [Ti(nacnac)(CH_2_^*t*^Bu)_2_]^+^ (1^+^), the ethylene insertion reaction is predicted to be less favorable compared to the α-H abstraction process, as revealed by calculations on the optimized structure (Fig. 9a). The calculated energy barrier for the α-H abstraction in d^0^ complex 1^+^ is Δ*H*^‡^_298_ = 29.7 kcal mol^−1^ and Δ*G*^‡^_298_ = 30.3 kcal mol^−1^, being slightly less than the one calculated for d^1^ complex 1. This is consistent with a stronger degree of π character in the Ti–C bonds (*θ*_NHO–C–Ti_ = 23.5° and 17.5° for the two Ti–C bonds) of 1^+^. For ethylene insertion involving 1^+^, the TS barrier is Δ*H*^‡^_298_ = 28.3 kcal mol^−1^ and Δ*G*^‡^_298_ = 41.7 kcal mol^−1^ with respect to the initial reagents. Despite having a stronger degree of π character of the Ti–C bonds, the TS energy of ethylene insertion is strongly increased by Δ*G* = + 8.0 kcal mol^−1^ for the d^0^ complex compared to the analogous d^1^ complex. This indicates that the unpaired electron in a singly occupied molecular orbital of complex 1 plays an important role in its reactivity towards ethylene insertion, significantly lowering the TS energy for d^1^ active species compared to similar d^0^ species.

For the neutral supported alkyl species in 1@Al_2_O_3-700_, the overall energy barriers for C_2_H_4_ insertion into the Ti–C bond are Δ*H*^‡^_298_ = 16.7 kcal mol^−1^ and Δ*G*^‡^_298_ = 29.7 kcal mol^−1^ and thus both are lower than those found for molecular complex 1. This is consistent with the high polymerization activity of 1@Al_2_O_3-700_.

Ethylene insertion into the Ti–C bonds for both 1 and 1@Al_2_O_3-700_ follows the formation of π-ethylene complexes. The structures of the complexes 1⋯C_2_H_4_ (Fig. 9b) and [(Al_s_O)Ti(nacnac)(CH_2_^*t*^Bu)(C_2_H_4_)] (1@Al_2_O_3-700_⋯C_2_H_4_, Fig. 9c) were obtained through DFT geometry optimizations along the TS imaginary modes (see ESI Part 2.6‡ for details of the optimization). These structures are close to those of the transition states for C_2_H_4_ insertion (see Fig. S13‡) with two CH_2_^*t*^Bu ligands being *trans* to each other for complex 1⋯C_2_H_4_ and with the CH_2_^*t*^Bu ligand being *trans* to the Ti–N bond of the nacnac ligand for complex 1@Al_2_O_3-700_⋯C_2_H_4_. In fact, for complex 1⋯C_2_H_4_, the calculated enthalpy and Gibbs free energy of formation with respect to the initial reagents (1 + C_2_H_4_) are Δ*H*^0^_298_ = 21.4 kcal mol^−1^ and Δ*G*^0^_298_ = 34.0 kcal mol^−1^ and very close to those of the TS (the corresponding TS energies are Δ*H*^‡^_298_ = 1.1 kcal mol^−1^ and Δ*G*^‡^_298_ = −0.3 kcal mol^−1^ with respect to the 1⋯C_2_H_4_ complex). The same is found for the 1@Al_2_O_3-700_⋯C_2_H_4_ π complex, with its CH_2_^*t*^Bu ligand being *trans* to the Ti–N bond of the nacnac ligand. With Δ*H*^0^_298_ = 16.4 kcal mol^−1^ and Δ*G*^0^_298_ = 28.2 kcal mol^−1^ the energy barriers remaining to reach the TS are almost zero and calculated to be Δ*H*^‡^_298_ = 0.3 kcal mol^−1^ and Δ*G*^‡^_298_ = 1.5 kcal mol^−1^. Therefore, the energy cost for ethylene polymerization is mostly due to the initial formation of the π complexes followed by an almost barrier-less insertion, which is again in agreement with the observed induction period. The relatively high barrier of formation of the π-ethylene complex 1⋯C_2_H_4_ could be overcome by elevated ethylene pressure and increased temperature; this is consistent with the high temperatures and pressures (*e.g.* 80 °C, 7 bar) required for efficient polymerization on 1 (see Fig. 2a).

For the calculated Ti(iv) model cationic complex [Ti(nacnac)(C_2_H_4_)(CH_2_^*t*^Bu)_2_]^+^ (1^+^⋯C_2_H_4_, Fig. 9d), the TS energy of ethylene insertion with respect to this complex is close to that of its d^1^ analogue 1⋯C_2_H_4_ (Δ*H*^‡^_298_ = 0.9 kcal mol^−1^ and Δ*G*^‡^_298_ = 2.4 kcal mol^−1^). Again, as soon as a π-ethylene complex is formed, the ethylene insertion into the Ti–C bond is nearly barrier-less; this behavior occurs in both d^1^ and d^0^ complexes. At the same time, while the structure is similar to that of the 1⋯C_2_H_4_ complex, the formation energy of 1^+^⋯C_2_H_4_ is much higher than that for 1⋯C_2_H_4_ (Δ*H*^0^_298_ = 27.4 kcal mol^−1^, Δ*G*^0^_298_ = 39.3 kcal mol^−1^). We thus further examine the π-ethylene complexes 1⋯C_2_H_4_, 1^+^⋯C_2_H_4_ and 1@Al_2_O_3-700_⋯C_2_H_4_*via* NBO analyses.

#### Back donation from the unpaired electron orbital and the enhanced π character in Ti–C bonds of the π-ethylene complexes support the “augmented” Cossee–Arlman polymerization mechanism

For both 1⋯C_2_H_4_ and 1@Al_2_O_3-700_⋯C_2_H_4_, the degree of the π character of the Ti–C bonds may be indirectly estimated through the corresponding Ti–C–H angles in the calculated structures of the complexes, which acts as a marker of the α-agostic C–H interaction. For the complex 1⋯C_2_H_4_, the corresponding acute angles are 85.73° for the shortened (2.188 Å) Ti–C bond and 82.04° for the elongated (2.239 Å) Ti–C bond. For 1@Al_2_O_3-700_⋯C_2_H_4_, the Ti–C–H angle is 90.30°. This points towards an increase of the π character in the metal–carbon bonds in the 1⋯C_2_H_4_ and 1@Al_2_O_3-700_⋯C_2_H_4_ complexes compared to the initial 1 and 1@Al_2_O_3-700_.

The degree of π character in Ti–C bonds of both the π complexes is further shown *via* the deviation of the corresponding NHO on C from the Ti–C axis, being *θ*_NHO–C–Ti_ = 31.3° for the shortened and 38.5° for the elongated Ti–C bonds of 1⋯C_2_H_4_, and *θ*_NHO–C–Ti_ = 26.1° for the Ti–C bond of 1@Al_2_O_3-700_⋯C_2_H_4_. For the Ti(iv) 1^+^⋯C_2_H_4_ complex, these values are *θ*_NHO–C–Ti_ = 25.6° and 39.7° for the two Ti–C bonds, being close to the ones for 1⋯C_2_H_4_. Therefore, a significant increase of the π character is observed for both d^1^ and model d^0^ π-ethylene complexes. These values are close to the ones previously found for the cationic Zr(iv) and Ti(iv) d^0^ complexes [Cp_2_MEt(C_2_H_4_)]^+^, with *θ*_NHO–C–Ti_ = 40.9° for M = Ti and *θ*_NHO–C–Ti_ = 40.4° for M = Zr.^35^ Note that all mentioned π-ethylene complexes, while having an enhanced degree of the π character in their Ti–C bonds, provide nearly barrier-less ethylene insertion with rather low calculated or reported^35^ TS energies. This indicates that ethylene insertion in neutral Ti(iii) alkyl species or cationic Ti(iv) and Zr(iv) alkyls depends on the extent of the π character in Ti–C bonds. Therefore, a strong π character is a general reason for the facile ethylene insertion in d^1^ and d^0^ metal alkyl complexes after the coordination of C_2_H_4_.

**Fig. 10:**
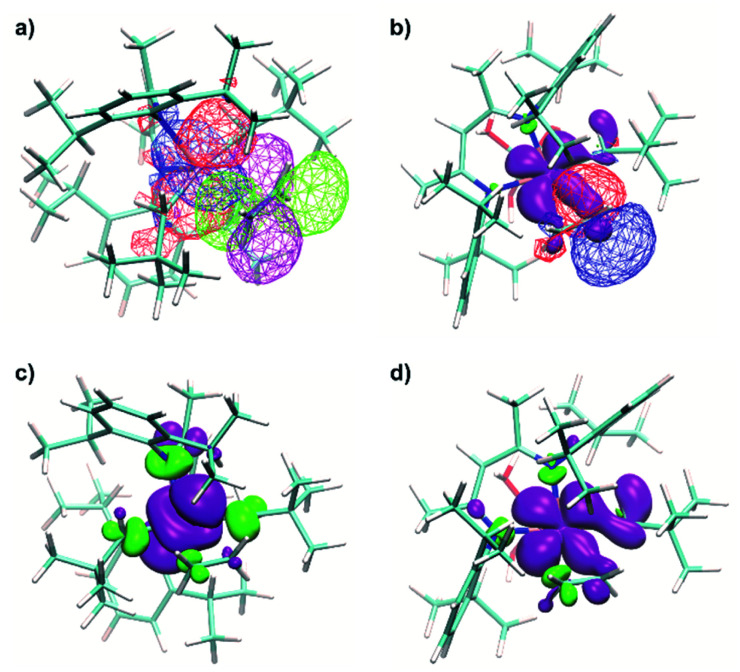
(a) Natural orbital, related to a SOMO (red for positive and blue for negative signs of the wavefunction), together with a natural orbital, related to π*(C_2_H_4_) (purple for positive and green for negative signs of the wavefunction) of 1⋯C_2_H_4_. (b) Calculated spin density distribution (purple for positive and green for negative, contour levels 0.2%), together with the natural orbital, related to π*(C_2_H_4_) (red for positive and blue for negative signs of the wavefunction), of the 1@Al_2_O_3-700_⋯C_2_H_4_ complex. (c) Calculated spin density distribution (purple for positive and green for negative signs, contour levels 0.1%) for the TS of ethylene insertion for 1. (d) Calculated spin density distribution (purple for positive and green for negative signs, contour levels 0.1%) for the TS of ethylene insertion for 1@Al_2_O_3-700_. The degree of delocalization of the unpaired electron could be estimated from the spin density distribution in the first coordination sphere of Ti, including ethylene.

It is noteworthy that for Ti(iii) d^1^ complexes 1 and 1@Al_2_O_3-700_ “back donation” of the unpaired electron from a SOMO to the π* orbital of coordinated C_2_H_4_ is also involved in the insertion process. These two orbitals have a constructive overlap, as revealed by the NBO analysis for complex 1⋯C_2_H_4_*via* the overlap of negative parts of the corresponding natural orbitals (Fig. 10a). This results in some weakening of the C=C double bond of the C_2_H_4_ ligand (C–C distance is 1.344 Å compared to the calculated value of 1.333 Å for free ethylene), together with a population of the π*(C_2_H_4_) orbital (see ESI Part 2.7‡). The NBO energetic analysis reveals the stabilization effect, caused by the presence of a π*(C_2_H_4_) orbital in 1⋯C_2_H_4_, of 30.0 kcal mol^−1^. At the same time, the stabilization effect, caused by the presence of the π*(C_2_H_4_) orbital in the d^0^1^+^⋯C_2_H_4_ complex, is 23.1 kcal mol^−1^. Therefore, the energy of the back donation of unpaired electron density in 1⋯C_2_H_4_ could be estimated through the difference of these two as a stabilization by 6.9 kcal mol^−1^, consistent with the difference of formation enthalpies Δ*H*^0^_298_ of 1⋯C_2_H_4_ and 1^+^⋯C_2_H_4_ of 6.0 kcal mol^−1^. This indicates that the back donation of the unpaired electron from the SOMO to the π* orbital of C_2_H_4_ is the main reason for relative stabilization of the π-ethylene complex 1⋯C_2_H_4_ compared to its d^0^ analogue 1^+^⋯C_2_H_4_. As the TS energies of ethylene insertion after the formation of π-ethylene complexes 1⋯C_2_H_4_ and 1^+^⋯C_2_H_4_ differ only by 2.7 kcal mol^−1^ (Δ*G*^‡^_298_ = −0.3 kcal mol^−1^ for 1⋯C_2_H_4_, compared to Δ*G*^‡^_298_ = 2.4 kcal mol^−1^ for 1^+^⋯C_2_H_4_), the principal reason for the lowering of the overall TS energy for d^1^ catalyst 1 by Δ*G* = 8.0 kcal mol^−1^ is the back donation of the unpaired electron to the π* orbital of C_2_H_4_.

An even higher degree of back donation to π*(C_2_H_4_) is observed for the 1@Al_2_O_3-700_⋯C_2_H_4_ complex, since a strong spin density transfer from the initial SOMO to π*(C_2_H_4_) is revealed by the calculated spin density distribution (Fig. 10b). Similar to the molecular 1⋯C_2_H_4_ complex, this leads to a weakening of the C=C double bond of the C_2_H_4_ molecule (C–C distance is 1.364 Å) and to the appearance of a bonding interaction between Ti in 1@Al_2_O_3-700_ and C_2_H_4_. In fact, NBO analysis, being one of the possible ways of representation of the electronic structure of 1@Al_2_O_3-700_⋯C_2_H_4_, shows a breaking of the π system of ethylene, followed by formation of a bonding set of natural orbitals, corresponding to a Ti–C(C_2_H_4_) bond (Fig. S14 and Table S2‡), and a partially occupied natural lone pair on the other carbon atom of C_2_H_4_, derived from π*(C_2_H_4_) (Fig. 10b). This indicates more favorable coordination of C_2_H_4_ to 1@Al_2_O_3-700_ where the stronger back donation in 1@Al_2_O_3-700_⋯C_2_H_4_ is caused by better orbital overlap between the initial SOMO and π*(C_2_H_4_). The better overlap between the SOMO and π*(C_2_H_4_) in the supported catalyst is mostly caused by the difference in geometry of the π-ethylene complexes induced by replacing one of the strong σ-donor alkyl ligands (CH_2_^*t*^Bu) by a weaker O anionic surface ligand OAl_s_. This is consistent with the lower formation energy of 1@Al_2_O_3-700_⋯C_2_H_4_ of Δ*H*^0^_298_ = 16.4 kcal mol^−1^ compared to Δ*H*^0^_298_ = 21.4 kcal mol^−1^ for complex 1 and with the ability of 1@Al_2_O_3-700_ to catalyze ethylene polymerization under milder conditions.

**Scheme 2:**
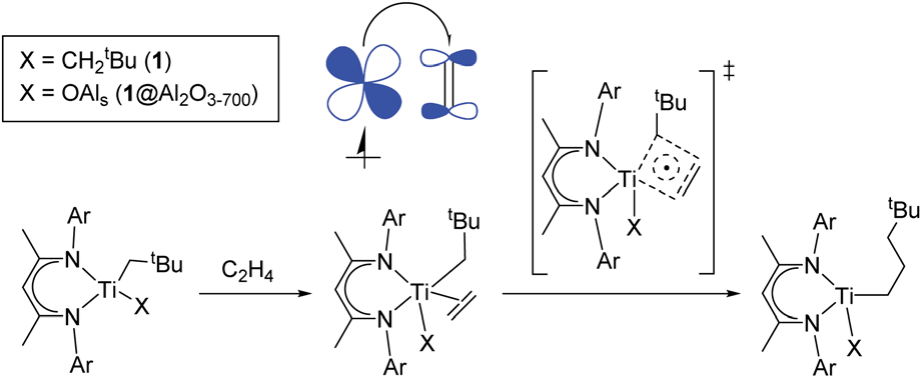
“Augmented” Cossee–Arlman mechanism of ethylene polymerization for 1 and 1@Al_2_O_3-700_. A partial electron transfer process from the SOMO to π*(C_2_H_4_) (“back donation”) is shown in the molecular orbital picture.

In fact, the unpaired electron in the 1@Al_2_O_3-700_⋯C_2_H_4_ complex appears to be strongly delocalized between the Ti d orbital, π*(C_2_H_4_) and σ*(Ti–C_CH2^*t*^Bu_), as revealed by the spin density distribution (Fig. 10b) and the occupancies of the related natural orbitals (see Table S2‡). The same delocalization is also found in the structure of the TS of ethylene insertion for both 1 and 1@Al_2_O_3-700_, as revealed by the calculated spin density distributions (Fig. 10c and d). This delocalization appears to be stronger for the TS for 1@Al_2_O_3-700_ (Fig. 10d), while being weaker for the TS for 1 (Fig. 10c). It appears that the unpaired electron, while weakening the C=C double bond of C_2_H_4_ and facilitating the formation of a Ti–C(C_2_H_4_) bond due to its presence at π*(C_2_H_4_), also favors the cleavage of the Ti–C bond of the CH_2_^*t*^Bu ligand by occupation of the σ*(Ti–C) orbital before ethylene insertion. These factors together facilitate the C_2_H_4_ insertion into the Ti–C bond, in addition to the previously mentioned factor of its π character, which is consistent with a lowered energy of the TS of ethylene insertion in 1⋯C_2_H_4_, compared to 1^+^⋯C_2_H_4_, by Δ*G* = 2.7 kcal mol^−1^. We, therefore, propose that the mechanism of ethylene polymerization for 1 and 1@Al_2_O_3-700_ catalysts involves the delocalization of the unpaired electron in the TS (Scheme 2).

In general, the mechanism of ethylene insertion in d^1^ Ti(iii) alkyl complexes 1 and 1@Al_2_O_3-700_ is determined by two key factors: the π character in Ti–C bonds of (CH_2_^*t*^Bu) ligands and the back donation of the unpaired electron. While the presence of the π character facilitates the insertion of ethylene into Ti–C bonds after its coordination, making the insertion in π-ethylene complexes nearly barrier-less at the TS, the back donation significantly lowers the formation energies of the π-ethylene complexes, which facilitates the overall reaction of C_2_H_4_ insertion. The delocalization of the unpaired electron in the TS structure, being noticeable for the systems with a high degree of back donation (*i.e.*1@Al_2_O_3-700_⋯C_2_H_4_), also has an effect on this reaction, favoring the cleavage of Ti–C and C=C bonds and slightly lowering the TS barrier. We denote this process (Scheme 2) as an “augmented” Cossee–Arlman mechanism, being essentially a [2σ + 2π + d^1^] cycloaddition involving a partially alkylidenic σ(Ti–C) bond and a π(C_2_H_4_) bond together with a delocalized d^1^ electron.

The described delocalization of the unpaired electron is likely an important feature in d^1^ systems able to polymerize olefins, with a degree of the back donation (electron density transfer) that depends on the overlap of the SOMO and π*(C_2_H_4_), which in turn depends on the geometry of the system. For instance, a higher polymerization activity towards styrene polymerization was observed experimentally for Cp*Ti(OCH_3_)_2_/MAO and Cp_2_TiCl/MAO catalytic systems compared to Cp*Ti(OCH_3_)_3_/MAO and Cp_2_TiCl_2_/MAO, respectively.^21^ It is likely that the Ti(iii) species, active towards styrene polymerization, show better performance compared to similar Ti(iv) systems due to a strong back donation (electron transfer), favored by the aromatic system of styrene. Therefore, under the same polymerization conditions, a d^1^ catalyst may be more active than a d^0^ catalyst of a similar structure. This finding also further suggests that Ti(iii) alkyl species have competent electronic structures to act as efficient polymerization catalysts and may indeed be active species in the classical Ziegler–Natta heterogeneous catalysts.

## Conclusions

In this work, we report the polymerization activity of molecular and the corresponding alumina-supported well-defined Ti(iii) neutral alkyl species prepared *via* surface organometallic chemistry. Both of them were characterized and studied in detail by pulse EPR spectroscopy, combined with DFT calculations. This approach enabled us to identify the prevalent conformation of the molecular complex [Ti(nacnac)(CH_2_^*t*^Bu)_2_] in a frozen toluene solution and to reveal the structure of the alumina-supported species that correspond predominantly to a neutral Ti(iii) alkyl compound, *i.e.* [(Al_s_O)Ti(nacnac)(CH_2_^*t*^Bu)]. To the best of our knowledge, these are the first examples of well-defined Ti(iii) alkyl species able to efficiently polymerize ethylene, producing ultra-high molecular weight polyethylene. The ethylene insertion into the Ti(iii)–C bond of [Ti(nacnac)(CH_2_^*t*^Bu)_2_] was further evidenced by EPR hyperfine spectroscopy (CHEESY-detected NMR), using isotope-labeled Ti(nacnac)(^13^CH_2_^*t*^Bu)_2_ in contact with C_2_H_4_.

These Ti(iii)-based polymerization pre-catalysts display α-agostic C–H bonds in their (CH_2_^*t*^Bu) ligands, supporting the presence of π character in the corresponding metal–carbon bonds.^35^ Such a π character was further supported by DFT calculations *via* NBO analysis. It is noteworthy that the presence of the half-filled d^1^ Ti orbital does not prevent α-agostic C–H bonding. After coordination of C_2_H_4_, the degree of π character in the Ti–C bonds of (CH_2_^*t*^Bu) ligands is significantly increased, which allows a nearly barrier-less insertion of C_2_H_4_ into the Ti–C bonds. Hence, the slow step is olefin coordination, consistent with the need for high pressure to carry out this reaction and with the observation of an induction period. The back donating interaction (electron transfer) between the SOMO and the π* orbital of C_2_H_4_ results in a significant lowering of the formation energies of π-ethylene complexes, which facilitates an overall reaction of ethylene insertion in these Ti(iii) systems. Due to the back donation, the unpaired electron could be delocalized between the Ti d orbital and π*(C_2_H_4_) and σ*(Ti–C) orbitals in both π-ethylene complexes and transition states, which also lowers the energy barriers for ethylene insertion. All these factors, which combine to give an “augmented” Cossee–Arlman mechanism, facilitate the overall reaction of C_2_H_4_ insertion into the Ti–C bond, making the ethylene polymerization in d^1^ metal complexes potentially more efficient than that in d^0^ complexes of a similar structure under the same conditions.

This study shows that neutral d^1^ Ti alkyl complexes are competent in ethylene polymerization, being favored by a combination of the π character in the Ti–C bonds and the back donation of the unpaired electron. These findings lend further support to the notion that d^1^ Ti-alkyls are possible active sites in the heterogeneous Ziegler–Natta polymerization catalysts.

## Author contributions

A. A. and F. A. contributed equally. A. A. carried out CW EPR and polymerization tests for 1, HYSCORE measurements, DFT computations, NBO analysis and wrote the manuscript. F. A. performed synthesis and characterization of 1, 1* and 1@Al_2_O_3-700_ and polymerization tests for 1@Al_2_O_3-700_. N. W. conducted CHEESY-detected NMR measurements. D. K. supervised the project. S. K. proposed the Ti(nacnac)(CH_2_^*t*^Bu)_2_ complex as a stable Ti(iii) alkyl compound to be tested for ethylene polymerization activity. G. J. and C. C. coordinated the project and provided guidance. All the authors revised the manuscript.

## Conflicts of interest

There are no conflicts to declare.

## Supplementary Material

SC-012-D0SC04436A-s001
