# Supplementary material for: Molecular and supported Ti(iii)-alkyls: efficient ethylene polymerization driven by the π-character of metal–carbon bonds and back donation from a singly occupied molecular orbital
Source: Chem Sci. 2020 Nov 11;12(2):780–92. doi: 10.1039/d0sc04436a (PMC8178971; doi:10.1039/d0sc04436a)
Supplement: SC-012-D0SC04436A-s001 [file SC-012-D0SC04436A-s001.pdf]

Electronic Supplementary Material (ESI) for Chemical Science.
This journal is © The Royal Society of Chemistry 2020

Supporting Information

# Molecular and Supported Ti(III)–Alkyls: Efficient Ethylene Polymerization from π-character of Metal-Carbon bonds and Back Donation from a Singly Occupied Molecular Orbital

Anton Ashuiev, Florian Allouche, Nino Wili, Keith Searles, Daniel Klose, Christophe Copéret and Gunnar Jeschke

**DOI: 10.1039/x0xx00000x**

## Table of Contents


1. Experimental procedures 2
2. Results and discussion 4
   2.1. Synthesis and characterization (**Figs. S1-S3**) 4
   2.2. Polymerization activity (**Fig. S4**) 6
   2.3. Detailed EPR characterization and evaluation of the structures of **1** and **1**@ $Al_2O_{3-700}$ (**Figs. S5-S10**) 7
   2.4. $^{13}C$ labelling as a tool to probe Ti-alkyl chains and polymerization mechanism (**Figs. S11-S12**) 12
   2.5. α-Agostic C-H interaction and π character of Ti-C bonds of **1** and **1**@ $Al_2O_{3-700}$ 15
   2.6. Olefin Polymerization Pathways with **1** and **1**@ $Al_2O_{3-700}$ (**Fig. S13**) 15
   2.7. Back donation from the unpaired electron orbital and the enhanced π character in Ti-C bonds of the π-ethylene complexes support "augmented" Cossee-Arlman polymerization mechanism (**Fig. S14**) 17
3. References 18


## Experimental Procedures

General procedures. All experiments were carried out under dry and oxygen-free argon atmosphere, using either standard Schlenk or glovebox techniques for organometallic synthesis. All syntheses were performed in pentane or THF. All samples for EPR spectroscopy were prepared in quartz tubes, using a flame sealing under high vacuum conditions ($10^{-5}$ mbar). For EPR measurements of frozen solutions, toluene was used as a good glass-forming solvent. Pentane and toluene were purified using double semi-permeable surface (SPS) alumina columns (MBraun, Garching, Germany), and were degassed using three freeze-pump-thaw cycles before being used. THF was distilled from Na/Benzophenone. For polymerization tests, ethylene was passed through activated 4 Å molecular sieves (Sigma-Aldrich, Buchs, Switzerland) and Cu Q5 catalyst (BASF, Ludwigshafen, Germany) to remove oxygen and water before utilization.
Elemental analysis. All elemental analyses were performed by the "Mikroanalytisches Labor Pascher" (Remagen, Germany).
NMR spectroscopy. All solution NMR spectra were recorded on a Bruker DRX 300 spectrometer (Bruker Biospin, Rheinstetten, Germany) in $C_6D_6$ at room temperature. The $^1H$ chemical shifts were referenced relative to the residual solvent peak.
Fourier transform Infrared Spectroscopy (FTIR). Transmission infrared spectra were recorded on a Bruker Alpha-T FT-IR spectrometer placed in a glovebox with oxygen-free argon atmosphere. The samples, taken as solid powders, were pressed into pellets; the FTIR spectra were measured in a range 4000 – 1100 $cm^{-1}$.
Scanning electron microscopy (SEM). SEM was done at Leo 1530 Gemini (Zeiss, Oberkochen, Germany) electron microscope with electron high-tension voltage of 1 kV and working distance of 5.6 mm.
Polymerization activity. The polymerization productivity of molecular complex Ti(nacnac)($CH_2{}^tBu$)$_2$ (**1**) was studied by examination of ethylene consumption at constant pressure and temperature with Biotage Endeavor system (Biotage, Uppsala, Sweden). For the alumina-grafted sample **1**@$Al_2O_{3-700}$, polymerization productivity was studied in a closed autoclave; the overall ethylene consumption was detected *via* a pressure change in the closed reactor after the reaction.
Size exclusion chromatography (SEC). The molecular weight distribution of polyethylene was determined with the method of size exclusion chromatography with a multi-angle light scattering detector (SEC-MALS). All SEC studies were performed by the "DSM Material Science Center" (DSM, Heerlen, Netherlands).
CW EPR spectroscopy. Room-temperature CW EPR spectra were recorded on a Bruker Elexsys E500 EPR spectrometer. Low-temperature CW EPR spectra were recorded at Bruker Elexsys E680 EPR spectrometer, equipped with a helium flow cryostat ESR900 (Oxford Instruments, Oxfordshire, UK). CW measurements were performed using a Bruker SHQ resonator, with a modulation amplitude of 0.1 mT (room-temperature CW) or 0.2 mT (low-temperature CW) and modulation frequency of 100 kHz. The lock-in amplifier time constant and conversion time were set to 81.92 ms and 327.68 ms, respectively. The correction of the magnetic field offset was done using DPPH (Sigma-Aldrich, Buchs, Switzerland) as a standard. All simulations of experimental CW EPR spectra were performed using least squares fitting in EasySpin[1] to determine the parameters of the *g* tensor.
HYSCORE spectroscopy. X-band HYSCORE measurements were performed on a Bruker Elexsys E680 EPR spectrometer using a Bruker MS3 split-ring resonator. Q-band HYSCORE measurements were performed on a Bruker Elexsys E580 spectrometer with a homebuilt 3 mm Q-band resonator.[2] For all HYSCORE measurements, the standard 4-pulse sequence π/2-τ- π/2-$t_1$- π-$t_2$- π/2- τ-echo with pulse lengths $t_{\pi/2}$ = 24 ns, $t_\pi$ = 16 ns was used. Time steps $t_1$ and $t_2$ were set to 8 ns. An eight-step phase cycle was used to remove unwanted echo contributions. For all X and Q band HYSCORE experiments, the repetition rate was set to 2 kHz. For X band HYSCORE, the length of HYSCORE traces was set to 1440 ns in both dimensions for all samples. For Q band HYSCORE, the length of traces was set to 2880 ns for **1** and to 1440 ns for **1**@$Al_2O_{3-700}$.
CHEESY-detected NMR spectroscopy. All Chirp Echo EPR SpectroscopY (CHEESY)-detected NMR measurements were performed in Q band on a homebuilt spectrometer based on a fast arbitrary waveform generator (AWG),[3] using a homebuilt 3 mm Q-band resonator.[2] For 1D CHEESY-detected NMR measurements, the 3-pulse sequence HTA-$\tau_1$-π/2$_{chirp}$-$\tau_2$-π$_{chirp}$ -$\tau_2$-echo (HTA = high turning angle) was used, with $t_{HTA}$ =20 µs, $\tau_1$ =5 µs, $t_{\pi/2}$ = 200 ns, $t_\pi$ = 100 ns, $\tau_2$ = 1 µs. Chirp pulses covered a range of 800 MHz and the frequency modulation was adjusted for the resonator profile. [3] A Fourier transform of the echo gives the EPR spectrum in frequency domain. The difference of these spectra with and without HTA pulse yields the nuclear spectrum, and the frequency of the HTA pulse is taken as the reference frequency when plotting the spectra. For 2D CHEESY-detected NMR measurements, the 4-pulse sequence π(sel)-$\tau_1$-HTA-$\tau_1$-π/2$_{chirp}$-$\tau_2$-π $_{chirp}$ -$\tau_2$-echo was used, with $t_{\pi(sel)}$ = 1 µs (Gaussian shape) and otherwise identical parameters as for the 1D measurements. The frequency of the first (selective) π pulse was varied in order to create the indirect

dimension of the 2D spectrum. The difference between the spectra with and without selective inversion yields the intensities of the 2D spectrum.[4]

Simulations of HYSCORE and CHEESY spectra. All numerical simulations of experimental HYSCORE spectra were performed in EasySpin[1] with the *saffron* function, which took as an input DFT computed parameters of hyperfine and quadrupole tensors for the two nitrogen nuclei and of hyperfine tensors for the two protons of each ($CH_2{}^tBu$) ligands. $^{27}$Al couplings for the sample of **1**@$Al_2O_{3\text{-}700}$ were obtained by a least squares fit of experimental HYSCORE spectra, using the program Hyscorean.[5] The interpulse delays τ were taken into account in all the simulations of HYSCORE spectra; the excitation bandwidths of microwave pulses were assumed to be infinite.

All simulations of experimental 1D CHEESY spectra were done using home-written functions, which take as an input the experimental *g* tensor for complex **1**, together with DFT computed *g* tensor orientation, and DFT computed parameters of hyperfine and quadrupole tensors for the two nitrogen nuclei, together with experimentally determined $^{13}$C hyperfine couplings (for the selectively $^{13}$C-labelled complex Ti(nacnac)($^{13}CH_2{}^tBu)_2$ (**1***)). The algorithm is based on previous work,[6,7] but the orientation selection was chosen to be governed by the HTA pulse instead of the detection sequence. The chirp pulses were not explicitly modeled.

DFT calculations of EPR parameters. All calculations of the parameters of hyperfine and quadrupole tensors were performed in ORCA 3[8] for crystal structures and geometry optimized models, using the unrestricted Kohn-Sham formalism with the functional PBE0[9], polarized triple-ζ def2-TZVPP[10] basis set for Ti and Al atoms and EPR-II[11] basis set for all other atoms. Increased integration grids (Grid6 in ORCA convention) and tight self-consistent field convergence (VeryTightSCF in ORCA) were used for the calculations of EPR parameters and elsewhere. The spin density distribution was visualized for all the studied paramagnetic structures using VMD.[12]

The *g* tensor for complex **1** was calculated using the same parameters as for the calculations of hyperfine and quadrupole tensors (PBE0 [9] functional, def2-TZVPP [10] basis set for Ti and EPR-II [11] basis set for all other atoms). However, the amount of exact exchange in the PBE0 functional was increased up to 50 % (ScalHFX = 0.5 in ORCA convention), as previously suggested.[13] Both one-electron and two-electron terms of the spin-orbit coupling operator were taken into account, with exact evaluation of Coulomb terms, treatment of exchange terms *via* one-center exact integrals (including spin-other orbit interaction) and introduction of DFT local correlation terms (SOCFlags 1,4,3,1 in %rel block in ORCA convention).

DFT geometry optimizations. All geometry optimizations were performed in ORCA 3[8], using the unrestricted Kohn-Sham formalism with PBE0,[9] together with Becke's three-center dispersion correction,[14] The COSMO[15] continuum solvation model was used for all the molecular complexes dissolved in toluene (COSMO(Toluene) in ORCA convention), while it was not applied for all the models of alumina-grafted materials. All optimizations were done in two steps, related to the two different levels of theory. In the first step, the RIJCOSX approximation[16] was used, together with an auxiliary basis set[17] def2-TZVPP/J (in ORCA 3 [8], def2-SVP/J = def2-TZVP/J = def2-QZVP/J [18]) and increased numerical precision of COSX integration (IntAccX 5,5,5; GridX 3,3,4 in ORCA convention). In the second step, no RIJCOSX approximation[16] was used with the full def2-TZVPP [10] basis sets for all atoms. For all optimizations, tight geometry convergence criteria (TightOpt in ORCA convention) and variable step size (Trust 0.05 in ORCA convention) were used. All final structures were proven to correspond to minima of the potential energy surfaces by the absence of imaginary frequencies in numerical frequencies calculations. All the structures were visualized using VMD.[12]

Exact Hessian matrices and numerical frequencies calculations. All numerical frequencies calculations and exact Hessian matrix evaluations were performed using the same parameters as for the second step of geometry optimizations, i.e. the def2-TZVPP [10] basis set for all atoms. Such calculations were also used to determine thermodynamical parameters $\Delta H^0_{298}$ and $\Delta G^0_{298}$ for all the ground state structures and to determine $\Delta H^{‡}_{298}$ and $\Delta G^{‡}_{298}$ for all the transition states.

Relaxed surface scans. All relaxed surface scans were performed with certain structural parameters being fixed (e.g. dihedral angles D(N-Ti-C-C), interatomic distances, etc.), while converging the structures to the energy minima for all the other degrees of freedom. The fixed parameters are varied along the selected scan range. All the scans were performed with the RIJCOSX approximation[16] and the def2-TZVPP/J auxiliary basis set.[17]

Transition state (TS) calculations. All TS calculations were done in two steps with the same parameters as the ones used for ground state geometry optimizations. All the TS calculations included a preliminary step of bringing the structures as close as possible to the expected TS *via* the relaxed surface scans. After this step, the exact Hessian matrix was calculated with the RIJCOSX approximation and the TS structure was obtained *via* the energy maximization along the imaginary mode and energy minimization along all the other degrees of freedom, using the same parameters as for the first stage of ground state optimizations (RIJCOSX approximation[16]; tight geometry convergence criteria). At the second stage, the TS structures were refined without the RIJCOSX approximation, using the def2-TZVPP[10] basis set for all atoms as well as the previously calculated exact Hessian matrix. The exact Hessian was then recalculated for the final TS structure without RIJCOSX approximation. The TS saddle point was verified *via* quasi-Newton step optimization (the Hessian matrices were set to unity) at both sides of the saddle point of the calculated imaginary mode, producing the reagents and the products of corresponding reactions.

Natural bond orbitals (NBO) analysis. The NBO analysis, including natural atomic orbitals (NAO), natural bond orbitals (NBO) and natural hybrid orbitals (NHO) analyses, was done with the program NBO 7.0 [19], while taking the electronic structure system from ORCA 4.[20] The Kohn-Sham orbitals were generated in ORCA 4, using the same parameters as the ones used for DFT calculations of EPR parameters, and then transferred to the NBO program (NBO keyword in ORCA convention). As the unrestricted NBO analysis was performed, two sets of natural orbitals were obtained for all studied structures, related to spin-α and spin-β molecular orbitals; the maximal occupation of each natural orbital appeared equal to 1. The obtained natural bond orbitals were visualized through the ORCA output using VMD.[12]

The degree of π character in Ti-C bonds was estimated in a Natural Hybrid Orbital (NHO) Directionality and Bond Bending analysis, provided by an NBO program.[19] The provided NHO deviation angles ("Dev" values in "NHO directionality and bond bending" section of the output of NBO program[19]) inform on how the localized hybrid orbitals are directed relative to the internuclear vector (here Ti-C). The directions of $sp^{\lambda}d^{\mu}$ NHO hybrid orbitals for each atom are determined numerically to correspond to maximum angular amplitudes

(for sp$^{\lambda}$ hybrid orbital, this direction corresponds to the vector describing its *p*-component).[21] The program compares this direction with the direction of the line of centers between the two nuclei to determine the "bending" of the bond, expressed as the deviation angle "Dev" in the NBO program. A pure σ-bond built from the orbitals directed along the internuclear axis will have a NHO deviation of 0°, whereas pure π-type orientation corresponds to 90°.[22] Thus, increasing values of NHO deviation are associated with increasing mixing with orbitals that are not along the Ti-C bond, being an indication of the partial π-character.[23] In this work, the deviation angle of C-based sp$^{\lambda}$ NHO orbitals, from which the Ti-C bonds are built, from the line of centers between Ti and C was used as an indicative of the partial π-character in the Ti-C bonds.
The NBO energetic analysis was performed by the deletion of the elements from the NBO Fock matrix, corresponding to the selected weakly occupied NBO orbitals, diagonalization of the new Fock matrix and passing the obtained density matrix to the SCF routines (ORCA) for a single pass through the SCF energy evaluator.[21] The energy contribution of the selected orbital is evaluated through the difference between the obtained and original SCF energies.

## Results and Discussion

### 2.1. Synthesis and characterization

<u>Preparation of complex **1**</u>. Ti(nacnac)($CH_2{}^tBu$)$_2$, i.e. **1**, was synthesized according to literature procedures[24] from Ti(nacnac)$Cl_2$ and Li($CH_2{}^tBu$) (see Scheme 1c, main text); in contrast to the literature procedures, pentane was used as solvent instead of $Et_2O$. Li($CH_2{}^tBu$) was prepared from neopentyl chloride (Sigma-Aldrich) and n-Butyllithium (Sigma-Aldrich). Ti(nacnac)$Cl_2$ was prepared from $TiCl_3(THF)_3$ (Sigma-Aldrich) and Li(nacnac). Nacnac ligand was synthesized *via* Schiff condensation from acetylacetone (Sigma-Aldrich) and 2,6-diisopropylaniline (Sigma-Aldrich) and further deprotonated with n-Butyllithium.
<u>NMR characterization of complex **1**</u>. Complex **1** was characterized with solution NMR, consistent with its previous characterization[24] (Fig. S1).

**Figure S1.** Liquid NMR spectrum of **1** in $C_6D_6$.

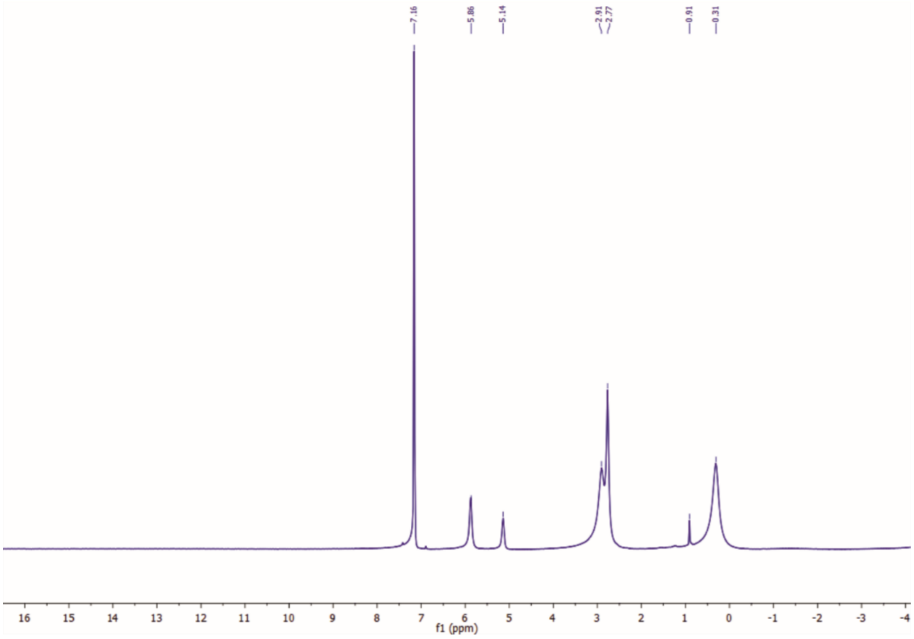


Experimental chemical shifts for **1**: δ (ppm) = 149.13; 41.04; 5.86; 5.14; 2.91; 2.77; 0.31. Low intensity peaks at 149.13 ppm and 41.04 ppm (not shown in Fig. S1) are very broad and of different phase with respect to main peaks shown in Fig. S1; they could be tentatively assigned to α-H of $CH_2{}^tBu$ ligands, being close to paramagnetic $Ti^{3+}$. Peak at 0.91 ppm corresponds to traces of neopentane $C(CH_3)_4$.
Previous NMR characterization[24]: δ = 5.84 (Δv = 20 Hz); 5.11 (Δv = 16 Hz); 2.94 (Δv = 105 Hz); 2.79 (Δv = 18 Hz); 0.30 (Δv = 49 Hz); -1.42 (Δv > 1000 Hz). The last peak is not observed on Fig. S1, being rather broad, and likely belongs as well to the α-H of $CH_2{}^tBu$ ligands.
<u>EPR characterization of complex **1**</u>. Complex **1** was characterized with both CW EPR of liquid solutions at room-temperature and frozen solutions at 10 K (Fig. S2).

**Figure S2.** a) Room-temperature EPR spectra of liquid solutions of **1** in $Et_2O$/pentane mixture (blue) and toluene (red). b) EPR spectra of frozen solutions of **1** in $Et_2O$/pentane mixture (blue) and toluene (red), 10 K. All field values are normalized by microwave frequency ratios.

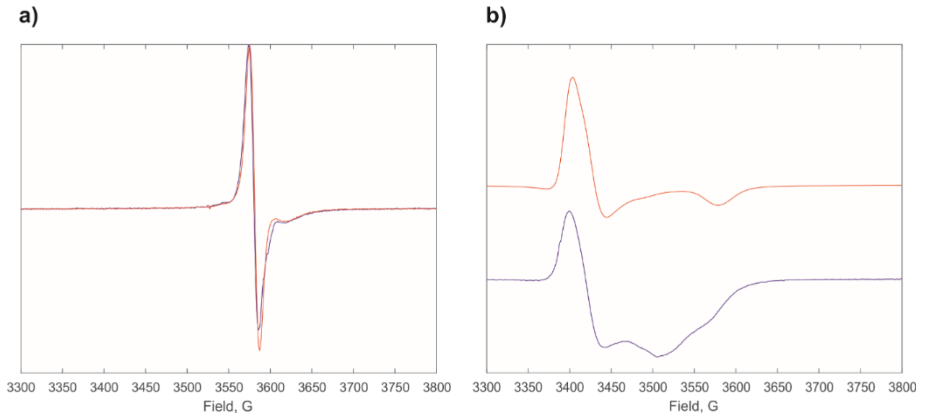


The room-temperature EPR spectra of **1** are identical for liquid solutions in, both, $Et_2O$/pentane mixture, used for the previous characterization[24], and toluene (Fig. S2, a). The determined isotropic $g_{iso}$ = 1.9748 ± 0.0089 (the given uncertainty indicates the FWHM width of Gaussian distribution of *g* value) is consistent with $g_{iso}$ = 1.97 of the previous characterization.[24] However, the CW EPR spectra of the same samples for frozen solutions at 10 K are different (Fig. S2, b). In an $Et_2O$/pentane mixture, a number of different conformations of **1** are produced, having smaller degree of *g* anisotropy than the conformation present in toluene solution. This explains the difference in *g* tensor values for **1** in current and previous[24] works. The currently determined $g_{exp}$ = [1.898 1.981 1.996] describes the CW EPR spectrum of **1** in frozen toluene solution at 10 K (see Fig. 1a of the main text), while the previously determined *g* = [1.955 1.963 1.963] describes CW EPR spectrum of **1** in frozen $Et_2O$/pentane mixture. Stronger anisotropy of the *g* tensor of **1** in toluene is supported by DFT calculations of the *g* tensor for the optimized structure of **1** in toluene (see Fig. 4a of the main text), which provides $g_{calc}$ = [1.9126 1.9703 1.9989].

Preparation of $Al_2O_{3\text{-}700}$: Alumina ($Al_2O_3$) Alu C (Evonik, Essen, Germany) was agglomerated to large particles by suspending it in distilled water, drying at 100°C, and sieving (grain size 250−400 µm) for easier handling. Sieved $Al_2O_3$ was calcined under a flow of synthetic air for 12 h at 500°C and then at 700°C for 10 h (referred to as $Al_2O_{3\text{-}700}$). The atmosphere was removed by vacuum ($10^{-5}$ mbar), while the reactor was still hot, and the alumina was stored in glovebox. It contained 0.10 mmol of OH per g, as measured by titration with $(C_6H_5CH_2)_2Mg(THF)_2$.

Synthesis of **1**@$Al_2O_{3\text{-}700}$: A solution of Ti(nacnac)($CH_2{}^tBu)_2$ (**1**) (61 mg, 0.10 mmol, 1 equiv.) in toluene (3 mL) was added to a slurry of 1.00 g of $Al_2O_{3\text{-}700}$ (0.10 mmol $Al_sOH$) in toluene (8 mL). After gentle stirring for 30 min at room temperature, the material **1**@$Al_2O_{3\text{-}700}$ was filtered, rinsed with toluene (4 × 5 mL) and pentane (1 x 5 mL), and dried under high vacuum ($10^{-5}$ mbar) at room temperature overnight. The amount of released neopentane was quantified by $^1H$ NMR of combined washings with a $Cp_2Fe$ internal standard.

Elemental analysis of **1**@$Al_2O_{3\text{-}700}$: The analysis was performed with a precision of 0.24±0.1 wt.% for Ti loading, the uncertainty of the analysis for lighter elements tends to increase. Elemental analysis of **1**@$Al_2O_{3\text{-}700}$ gives 0.24 wt% Ti, 2.06 wt% C, 0.20 wt% N and 0.24 wt% H, corresponding to 41.0 ± 1 C/Ti (34 expected), 3.4 ± 1 N/Ti (2 expected) and 57.0 H/Ti (52 expected).

Fourier transform Infrared Spectroscopy (FTIR). Transmission FTIR spectra were recorded in oxygen-free argon atmosphere for $Al_2O_{3\text{-}700}$ and **1**@$Al_2O_{3\text{-}700}$ (Fig. S3, red and purple, respectively). The IR lines of initial isolated hydroxyl groups on the $Al_2O_{3\text{-}700}$ surface at 3600 – 3810 $cm^{-1}$ (Fig. S3, red) disappeared after the grafting. For the grafted **1**@$Al_2O_{3\text{-}700}$ (Fig. S3, purple), IR lines with ν = 3081, 3064, 3025, 2968, 2874, 1595, 1531, 1495, 1465, 1437, 1389, 1377, 1365, 1317, 1278, 1255, 1230, 1180 and 1164 $cm^{-1}$ were observed, together with a broad line from 3420 to 3760 $cm^{-1}$, associated with remaining hydroxyl groups interacting with the grafted Ti organometallic fragments. The appearance of C-H signals at 2820 – 3080 $cm^{-1}$ indicates the presence of alkyl groups in **1**@$Al_2O_{3\text{-}700}$ material.

**Figure S3.** FTIR transmission spectra of $Al_2O_{3\text{-}700}$ (red), **1**@$Al_2O_{3\text{-}700}$ (purple) and **1**@$Al_2O_{3\text{-}700}$ contacted with 180 mbar of ethylene for 2 minutes (dark blue).

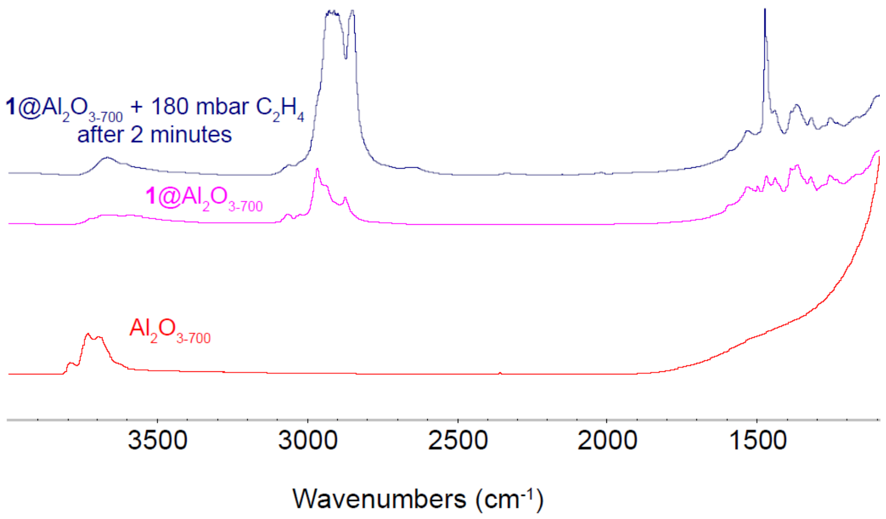


**2.2. Polymerization activity**

The material **1**@$Al_2O_{3\text{-}700}$ was contacted with 180 mbar of ethylene for 2 minutes. The formation of a polyethylene (PE) layer was observed by SEM (see Fig. 2c,d of main text) and by the intensity of C-H lines in FTIR spectrum (Fig. S3, dark blue), which increases after 2 minutes of contact with ethylene and reaches detector's saturation.

For the polymerization activity tests, the samples of molecular complex **1** (5 mM solution in toluene) were treated for 2 hours with 7 bars, 4 bars and 1 bar of $C_2H_4$ at 100 ˚C and with 7 bar of $C_2H_4$ at 80 ˚C, 90 ˚C and 100 ˚C. The ethylene consumption was recorded from the moment of application of $C_2H_4$ pressure; the reactors reached the set temperatures in ca. 10 minutes. For the sample of **1**@$Al_2O_{3\text{-}700}$, ethylene consumption was detected *via* a pressure change in a closed reactor at 50 ˚C for 16 hours with 6 bar initial pressure of ethylene.

In the absence of ethylene, molecular complex **1** is stable in 5 mM toluene and benzene solutions when heated to 80 ˚C for 2 hours. After the heating, no new dia- or paramagnetic species had appeared in the solution, as shown by NMR (Fig. S4, a) and EPR (Fig. S4, b) spectra.

**Figure S4.** a) Room-temperature EPR spectra of liquid solutions of **1** in $Et_2O$/pentane mixture (blue) and toluene (red). b) EPR spectra of frozen solutions of **1** in $Et_2O$/pentane mixture (blue) and toluene (red), 10 K. All field values are normalized by microwave frequency ratios.

a)

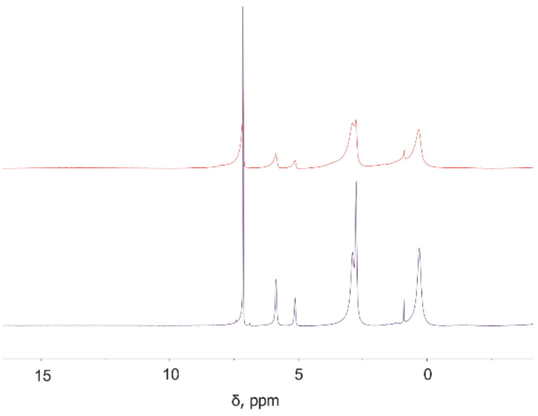


b)

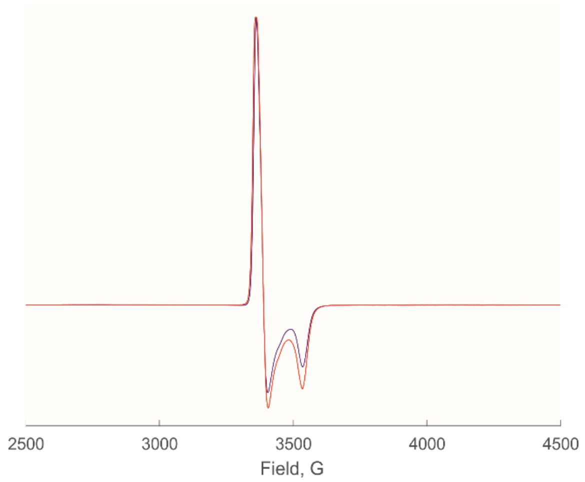


**2.3. Detailed EPR characterization and refinement of the structures of 1 and 1@$Al_2O_{3\text{-}700}$**

X band HYSCORE spectra with three τ values. For both samples **1** and **1**@$Al_2O_{3\text{-}700}$, the X-band HYSCORE spectra were measured with three different interpulse delays, τ = 128 ns, 160 ns and 224 ns to avoid loss of spectral information due to blind spots. The sum spectra are shown in Fig. S5. The spectra with τ = 128 ns contain all peak patterns present in the τ-summation spectra, except for the $^1H$ matrix peak. Therefore, one-τ HYSCORE spectra with τ = 128 ns can be used for our purposes without loss of significant spectral information, in particular when numerical simulations are used that take the interpulse delay τ into account. Matrix peak suppression at this interpulse delay is actually beneficial for observing the peaks of interest.

**Figure S5.** a) HYSCORE spectra of toluene solution of **1**, 10 K, τ = [128, 160, 224] ns. b) HYSCORE spectra of solid powder of **1**@$Al_2O_{3\text{-}700}$, 10 K, τ = [128, 160, 224] ns.

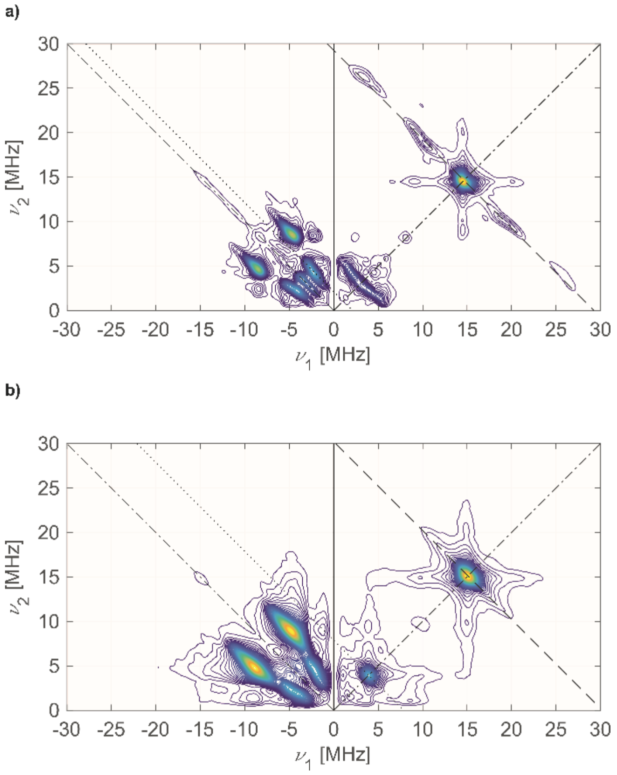


Crystal structure of **1**. Having performed the hyperfine and quadrupole tensor calculations for the crystal structure of **1** (Fig. S6, a), we found that the $^{1}H$ hyperfine couplings were not in agreement with the experimental HYSCORE spectrum (Fig. S6, b). Therefore, a further optimization of the crystal structure was needed in order to find an explicit model of **1** in toluene solution.

**Figure S6.** a) Crystal structure of **1**.[24] b) HYSCORE spectrum of **1**, τ = 128 ns, 10 K (blue) and simulation based on the calculated hyperfine and quadrupole tensors for the crystal structure of **1**.

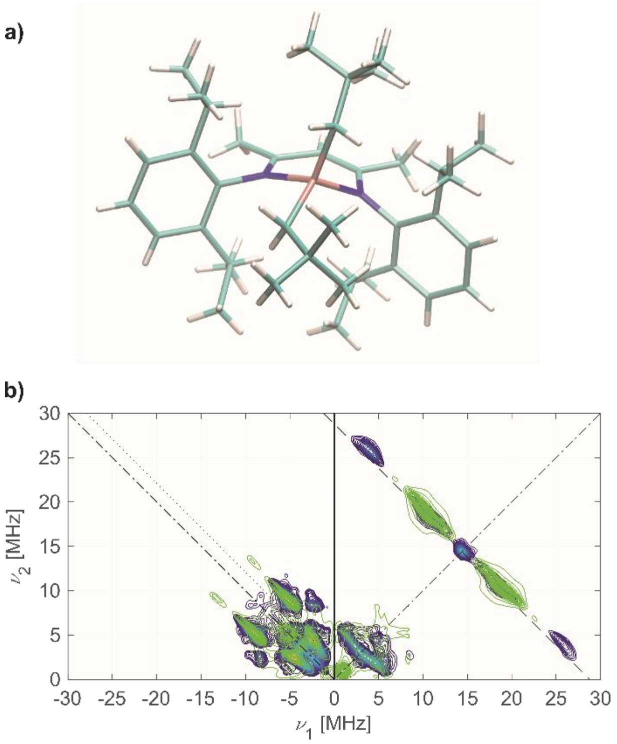


Dependence of EPR parameters for **1** on small structural changes. The parameters of hyperfine and quadrupole tensors of the EPR active nuclei of **1** are highly sensitive to small structural changes. As an example, we tested the influence of rotation of one of the $CH_2{}^{t}Bu$ ligands of **1** (perpendicular to the axial plane) around the Ti-C bond on the DFT-calculated EPR parameters. For such a rotation, a relaxed surface scan was performed, using the dihedral angle D(N-Ti-C-C) as a variable parameter. The relaxed surface

scan revealed two potential energy minima, corresponding to the two distinct conformations of **1** in toluene solution (Fig. S7, a). These conformations differ both by energy (by 0.72 kcal·mol$^{-1}$) and by calculated EPR parameters (Fig. S7, b & c). Both crystal[24] and optimized (vide infra) structures of **1** have the dihedral angle D(N-Ti-C-C) values close to the one corresponding to the lowest potential minimum on Fig. S7, a (58.08˚ and 66.01˚, respectively, compared to 67.67˚ on Fig. S7, a).

**Figure S7.** a) Change of molecular energy due to the rotation of one of $CH_2$$^t$Bu ligands of **1** in dependence on dihedral angle D(N-Ti-C-C). The dihedral angle was varied in a range of 145˚ width, being enough to reveal two distinct conformations of **1**. b) Change of the isotropic part of $^1$H hyperfine tensors for all 4 α-H atoms of $CH_2$$^t$Bu ligands of **1** due to the rotation of one of $CH_2$$^t$Bu ligands. The 1$^{st}$ and the 2$^{nd}$ α-H atoms belong to the rotating $CH_2$$^t$Bu ligand. c) Change in calculated double-quantum (DQ) $^{14}$N frequencies of both nitrogen atoms of **1** due to the rotation of one of $CH_2$$^t$Bu ligands. The frequencies were calculated for the field position used in Q-band CHEESY-detected NMR measurements of **1***, i.e. 1283.4 mT.

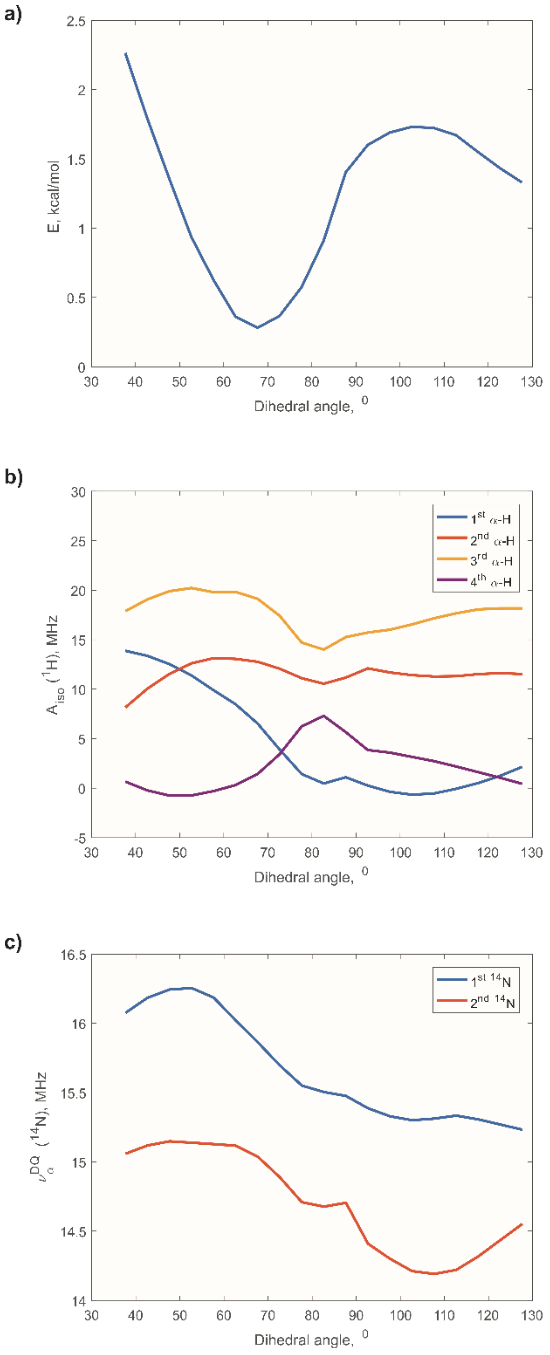


This rotation appeared to have a tremendous effect on the calculated isotropic part of the $^1$H hyperfine tensors of α-H atoms of both $CH_2$$^t$Bu ligands (Fig. S7, b). Such a rotation also affects the EPR parameters of the two nitrogen atoms of (nacnac) ligand, e.g. the calculated double-quantum (DQ) $^{14}$N frequencies (Fig. S7, c). The dependence of calculated hyperfine and quadrupole tensors of EPR active nuclei in $Ti^{3+}$ coordination sphere on even small structural changes allows us to use DFT-based simulations of experimental HYSCORE spectra as a tool for precise evaluation of molecular structures of **1** and **1**@$Al_2O_{3\text{-}700}$.

Optimized structure of **1**. A geometry optimization of the crystal structure of **1** was performed. The obtained structure (see Fig. 4a of the main text) appeared to provide the correct set of parameters of hyperfine and quadrupole tensors for the two nitrogen atoms of

(nacnac) ligand and the four α-H atoms of $CH_2{}^tBu$ ligands, which is able to simulate completely the experimental X band HYSCORE spectrum (see Fig. 4b of the main text). The same set of parameters of hyperfine and quadrupole tensors for the $^{14}N$ nuclei is also able to simulate reasonably the experimental Q-band HYSCORE spectrum for the selectively $^{13}C$-labelled complex Ti(nacnac)($^{13}CH_2{}^tBu$)$_2$ (**1***) (Fig. S8). This further supports the structure, shown in Fig. 4a, as the prevalent conformation of **1** in toluene solution and ensures that both prepared molecular complexes **1** and **1*** possess the same conformation. Therefore, the optimized conformation of **1**, shown in Fig. 4a, seems to be a general structure of Ti(nacnac)($CH_2{}^tBu$)$_2$ in toluene solutions independent from the isotope labeling or preparation batch.

**Figure S8.** a) Experimental Q-band HYSCORE spectrum of **1*** (τ = 200 ns), measured at the field position corresponding to the maximum of echo intensity. b) Q-band HYSCORE spectrum of **1*** (blue) and its simulation (green) based on the calculated hyperfine and quadrupole tensors for the optimized structure of **1**.

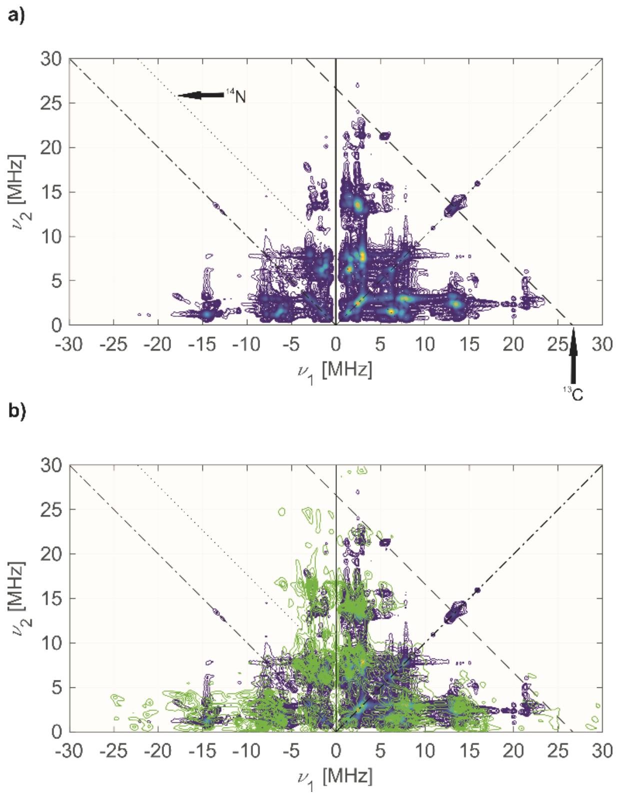


Cationic model of **1**@$Al_2O_{3-700}$. One of the possible structures for the **1**@$Al_2O_{3-700}$ species is represented by a model, corresponding to [Ti(nacnac)($OAl_S$)]$^+$···[($CH_2{}^tBu$)$Al_S$]$^-$ cationic species (Fig. S9, a), where the transfer of the $CH_2{}^tBu$ ligand to the Lewis acidic Al site of the $Al_2O_{3-700}$ surface occurs. To explore the possibility of such a transfer, we designed a model for the cationic species where the $CH_2{}^tBu$ ligand is as close as possible to the $Ti^{3+}$ center, with one bridging oxygen atom between Ti and ($CH_2{}^tBu$)$Al_S$. At the same time, two surface oxygen atoms $OAl_S$ are required to fill the coordination sphere of [Ti(nacnac)($OAl_S$)]$^+$ in order to produce stable four-coordinated Ti centers. This implies a presence of three $Al_s$ atoms in the cluster which, together with hydroxyl groups and coordinated water molecules added for charge and valence compensation, results in the model shown on Fig. S9, a.

However, for all the $^1H$ nuclei of such a model, the calculated isotropic and dipolar parts of hyperfine tensors are too small to simulate the experimental HYSCORE spectrum. The maximal calculated isotropic $^1H$ hyperfine coupling of $a_{iso}$ = 1.65 MHz ($a_{dip}$ = [-0.69 -0.59 1.29] MHz), corresponding to one the protons of $–CH_3$ groups of (nacnac) ring, is too small to simulate the experimental $^1H$ couplings (Fig. S9, b). All the protons of the $CH_2{}^tBu$ ligand, transferred to $Al_2O_{3-700}$ surface, have calculated isotropic $^1H$ hyperfine couplings less than 0.016 MHz, with maximal dipolar contributions of $a_{dip}$ = [-0.84 -0.86 1.70] MHz for one of the α-H atoms. This is much smaller than required for the simulation of $^1H$ hyperfine couplings in the experimental HYSCORE spectrum of **1**@$Al_2O_{3-700}$ (see Table 1 of the main text).

**Figure S9.** a) DFT-optimized model for cationic [Ti(nacnac)($OAl_S$)]$^+$···[($CH_2{}^tBu$)$Al_S$]$^-$ species. b) X-band HYSCORE spectrum of **1**@$Al_2O_{3-700}$ (blue) and its simulation (green), based on the calculated hyperfine tensor for the two $^{14}N$ nuclei and the most strongly coupled $^1H$ within the optimized model. The $^1H$ hyperfine couplings for the hydrogen atoms of OH and $H_2O$ groups in ($OAl_s$) cluster were not taken into account, as these groups are added for charge and valence compensation and are not actually present in **1**@$Al_2O_{3-700}$.

a)

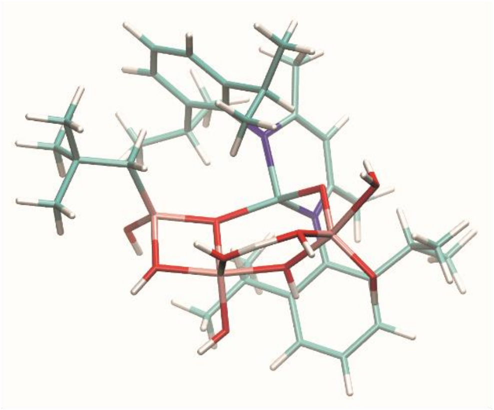

b)

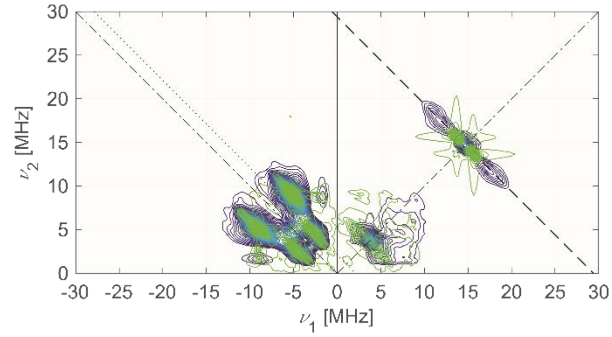


On the other hand, the calculated $^{14}$N hyperfine and quadrupole tensors are good enough to simulate experimental $^{14}$N peaks for both cationic and neutral models (see Fig. S9, b, and Fig. 5b of the main text). Therefore, we do not strictly exclude the presence of cationic species [Ti(nacnac)(OAl$_S$)]$^{+}$···[(CH$_2$$^t$Bu)Al$_S$]$^{-}$ at the $Al_2O_{3\text{-}700}$ surface after the grafting. However, strong intensity of elongated $^1$H ridges in experimental HYSCORE spectrum of **1**@$Al_2O_{3\text{-}700}$, which is not predicted by the cationic model, implies that such cationic species are not the major fraction of Ti(III) species on $Al_2O_{3\text{-}700}$ surface after the grafting. In fact, both **1** in toluene and **1**@$Al_2O_{3\text{-}700}$ produce ultra-high molecular weight polyethylene. This indicates that ethylene polymerization on **1** and **1**@$Al_2O_{3\text{-}700}$ most likely occurs by the same mechanism, i.e. *via* the $C_2H_4$ insertion into the Ti-C bond (vide infra), for which the neutral Ti(nacnac)(OAl$_s$)(CH$_2$$^t$Bu) species are required. A rather high polymerization activity for **1**@$Al_2O_{3\text{-}700}$ indicates the presence of a large number of Ti(nacnac)(OAl$_s$)(CH$_2$$^t$Bu) centers, which is consistent with neutral Ti(III) alkyls being the predominant species of **1**@$Al_2O_{3\text{-}700}$.
Neutral model of **1**@$Al_2O_{3\text{-}700}$. For the neutral Ti(nacnac)(OAl$_s$)(CH$_2$$^t$Bu) model (see Fig. 5a of the main text), $^{27}$Al hyperfine and quadrupole couplings were determined directly from experimental data. The $^{27}$Al couplings appear only as a matrix peak in X-band HYSCORE of **1**@$Al_2O_{3\text{-}700}$ (see Fig. 5b of the main text). However, they are resolved in Q-band HYSCORE (Fig. S10, a), which allows to extract the $^{27}$Al couplings through its numerical simulation via a least squares fit in Hyscorean[5] (Fig. S10, b).

**Figure S10.** a) Experimental Q-band HYSCORE spectrum of **1**@$Al_2O_{3\text{-}700}$ ($\tau$ = 294 ns), measured at the field position, corresponding to the maximum intensity of the echo-detected EPR spectrum. b) Q-band HYSCORE spectrum of **1**@$Al_2O_{3\text{-}700}$ (blue) and simulation (red), based on least squares fitting of $^{27}$Al peaks.

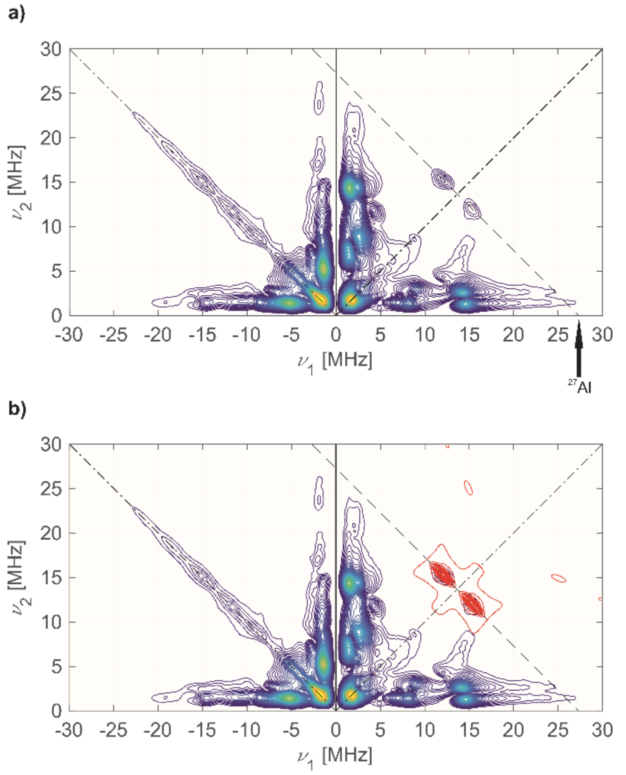


Determined EPR parameters for $^{27}Al$: $a_{iso}$ = 2.68 MHz; $a_{dip}$ = [-2.23; 3.56; -1.34] MHz; Q = 7.66 MHz (η = 0.01).
The thus-obtained $^{27}Al$ couplings, together with DFT-computed $^{14}N$ couplings, reasonably simulate the whole Q-band HYSCORE spectrum of **1**@$Al_2O_{3\text{-}700}$ (see Fig. 5c of the main text). A relatively small $^{27}Al$ quadrupole coupling is consistent with a highly symmetric coordination sphere of $Al_s$ atoms in Ti(nacnac)(O$Al_s$)($CH_2{}^tBu$), i.e. either with a tetrahedral or an octahedral surrounding of $Al_s$.
Comparison of g tensor principal values of **1** and **1**@$Al_2O_{3\text{-}700}$. The DFT-computed *g* tensor principal values for molecular complex **1** in frozen toluene were *g* = [1.9126 1.9703 1.9989], while for the neutral species of **1**@$Al_2O_{3\text{-}700}$ (Fig. 5a) they were *g* = [1.9354 1.9683 1.9892]. At the same time, the experimental *g* tensor principal values are *g* = [1.898±0.023 1.981±0.016 1.996 ± 0.012] for molecular complex **1** and *g* = [1.880±0.087 1.970±0.049 1.984±0.016] for **1**@$Al_2O_{3\text{-}700}$. While the large linewidth on $g_z$ orientation of **1**@$Al_2O_{3\text{-}700}$ prevents from making a direct comparison of this value between the two Ti(III) species, the tendency of decreasing of $g_{x,y}$ principal values, predicted by DFT, is also found for experimental values obtained from CW EPR. Note, however, that the trend is only of the same magnitude as the deviation between experimental and DFT-predicted values.

### 2.4. $^{13}C$ labelling as a tool to probe Ti-alkyl chains and polymerization mechanism

Observation of $^{13}C$ hyperfine couplings in **1*** by common hyperfine techniques. The observation of $^{13}C$ hyperfine couplings for directly coordinated $Ti^{3+}$-C atoms in **1** and **1**@$Al_2O_{3\text{-}700}$ systems appeared to be difficult, and is further complicated by the presence of a number of single- and multiple-quantum $^{14}N$ transitions, which have transition frequencies close to $\omega_{\alpha,\beta}$ ($^{13}C$). In fact, the $^{13}C$ signals for the selectively $^{13}C$-labelled complex Ti(nacnac)($^{13}CH_2{}^tBu$)$_2$ (**1***), corresponding to A($^{13}C$) = 16 MHz, could be observed in Q-band HYSCORE (Fig. S11, a), as well as in Q-band Davies ENDOR (Fig. S11, b). These weak signals (marked with arrows on Fig. S11) were confidently assigned to $^{13}C$ hyperfine couplings only after 1D & 2D CHEESY-detected NMR spectra became available (see Fig. 6 of the main text).

**Figure S11.** a) Experimental Q-band HYSCORE spectrum of **1*** (τ = 200 ns), measured at the field position, corresponding to the maximum of the echo-detected EPR spectrum. b) Q-band Davies ENDOR spectrum of **1*** ($t_\pi$ = 256 ns), measured at the field position, corresponding to the maximum of the echo-detected EPR spectrum. Signals, corresponding to $\nu_{\alpha,\beta}$($^{13}C$), are marked with red arrows.

a)

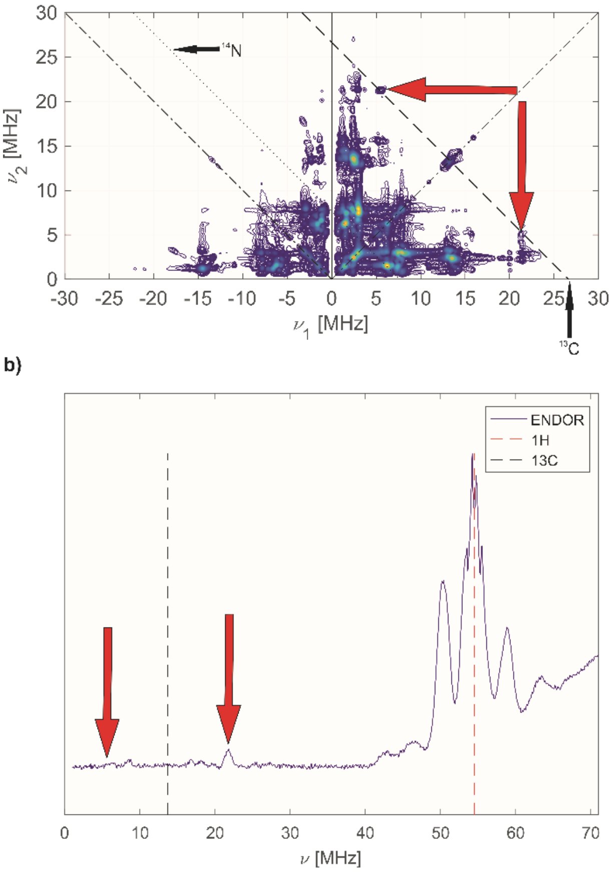


Both Q-band HYSCORE and Davies ENDOR (Fig. S11) show peaks corresponding to the $^{13}C$ hyperfine coupling $A_{exp}(^{13}C)$ = 16.0 MHz first determined from the CHEESY-detected NMR spectra. This value is reasonably close to the DFT-predicted $^{13}C$ couplings with absolute values of isotropic hyperfine couplings ($a_{iso}$ = -20.51 MHz, $a_{dip}$ = [1.26 0.54 -1.80] MHz and $a_{iso}$ = -21.69 MHz, $a_{dip}$ = [1.52 0.50 -2.01] MHz for the two $^{13}C$ nuclei). However, the quality of DFT calculations of EPR parameters for $^{13}C$ nuclei directly bonded to $Ti^{3+}$ appears to be worse than for the $^{14}N$ and $^{1}H$ nuclei.

Probing the polymerization mechanism with quantitative 1D CHEESY-detected NMR. For both samples **1** and **1**@$Al_2O_{3\text{-}700}$, the direct polymerization of $^{13}C$-labelled $^{13}C_2H_4$ (Scheme S1, b) does not lead to detectable $^{13}C$ hyperfine couplings by any of the used EPR hyperfine methods, including X- and Q-band 3-pulse ESEEM and 4-pulse HYSCORE, Q-band 5-pulse ESEEM, Q-band 1D- and 2D CHEESY-detected NMR together with standard 1D EDNMR, and Q-band Davies ENDOR. Therefore, the opposite reaction, i.e. $^{13}C$-labelled complex **1*** with non-labelled $C_2H_4$ was performed (Scheme S1, a), together with the observation of changes in intensity of $^{13}C$ signals of **1*** by CHEESY-detected NMR spectroscopy.

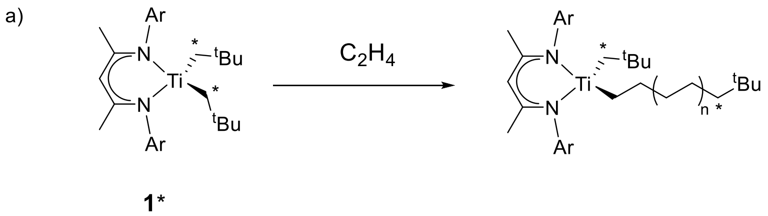


**1**

**Scheme S1.** Two possible isotope labelling schemes for the studies of the polymerization mechanism. $^{13}C$ atoms are marked with asterisks.

A 2 mM toluene solution of molecular complex **1*** and a 2 mM benzene solution of the same complex after the contact with ethylene (1000 equivalents, 2 hours, 80 ˚C under reflux) were sealed in 3 mm quartz tubes. A same sample height of 8 mm was used, which is sufficient to fill the active zone of the Q-band home-built resonator. For both samples, the profile of microwave absorption of the resonator ("mode picture") is nearly the same (Fig. S12). This allows to perform 1D CHEESY-detected NMR measurements at the same microwave frequency and field position for both samples and ensures nearly the same power of chirp microwave pulses along the scanning range. Although the absolute echo intensities for both samples may still be slightly different, the CHEESY-detected NMR signals, being essentially the ratio of the spectra with and without HTA pulse, provide sufficiently accurate quantification of the relative amount of EPR active nuclei in the Ti(III) coordination sphere before and after the reaction with $C_2H_4$.

**Figure S12.** Microwave absorption profiles of a home-built 3 mm Q-band resonator,[2] measured through the dependence of the electron nutation frequency $\nu_1$ on the microwave frequency $\nu_{mw}$ for the samples of molecular complex **1*** (black) and molecular complex **1*** after the contact with ethylene (red).

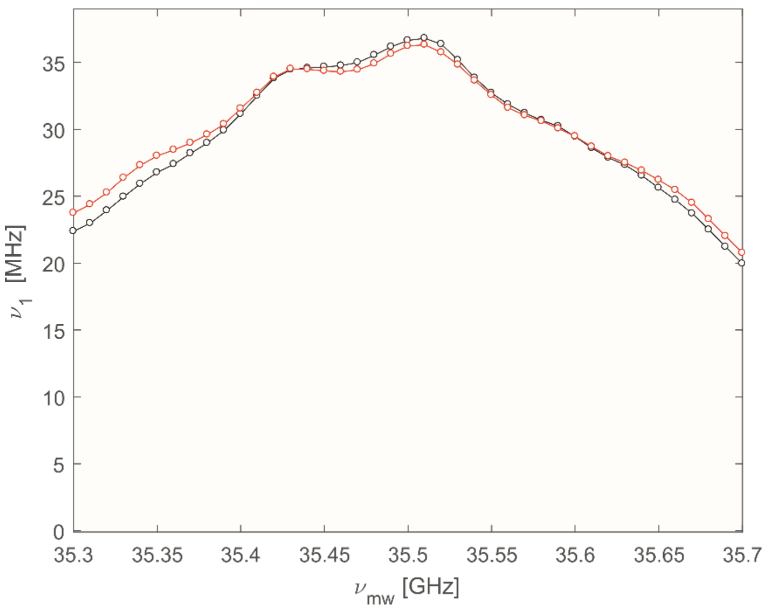


Indeed, the intensities of $^{14}N$ double-quantum transitions (around 14 MHz) in experimental 1D CHEESY-detected NMR spectra for the samples before and after ethylene polymerization (see Fig. 7b, top of the main text) are the same without any normalization applied. This is in a quantitative agreement with the presence of the same quantity of $^{14}N$ in the $Ti^{3+}$ coordination sphere for both samples of the same concentration. As the intensity of the $^{13}C$ signals, together with ($^{13}C$+$^{14}N$) combination lines, decreases in the same pair of spectra, we can safely conclude that $^{13}C$ nuclei are removed from the $Ti^{3+}$ coordination sphere during ethylene polymerization. As we know that the double-quantum $^{14}N$ frequencies are affected even by a small structural changes in **1** or **1***, such as the rotation of $CH_2{}^tBu$ ligands (see Fig. S7, c), we can safely exclude any larger changes in the coordination environment, including the elimination of $^{13}CH_2{}^tBu$ ligands (e.g. *via* α-H abstraction, hydrolysis, C-H activation in isopropyl groups of (nacnac) ligand, etc.). Thus, the most probable explanation of experimentally observed changes in 1D CHEESY-detected NMR spectra is the ligand exchange of $^{13}CH_2{}^tBu$ to $(CH_2CH_2)_{n-1}(^{13}CH_2{}^tBu)$ with preservation of the initial structure and conformation of **1*** (see Fig. 4a of the main text). This corresponds to the ethylene insertion into the Ti(III)-C bond.

Simulation of 1D CHEESY-detected NMR spectra. The simulation of the 1D CHEESY-detected NMR spectrum of **1**, governed by $^{14}N$ hyperfine and quadrupole couplings, was performed with DFT-calculated EPR parameters for $^{14}N$ nuclei (full hyperfine and quadrupole tensors, including tensor orientations, were used), based on the optimized structure of **1** (Fig. 4a of the main text). For the simulation of 1D CHEESY-detected NMR spectrum of **1***, determined by $^{13}C$ hyperfine and $^{14}N$ hyperfine and quadrupole couplings, the full $^{13}C$ hyperfine tensor was added. As the DFT-calculated EPR parameters for $^{13}C$ nuclei do not exactly fit the experimentally determined $A_{exp}(^{13}C)$ = 16.0 MHz, we used the experimental value as the isotropic part of $^{13}C$ hyperfine tensor in the simulation. However, as DFT predicts a negative sign for $a_{iso}(^{13}C)$, and the sign of hyperfine coupling is not determined in 1D CHEESY-detected NMR spectra, we assign the negative sign to the experimental hyperfine coupling to get $a_{iso}(^{13}C)$ = -16.0 MHz for both $^{13}C$ nuclei. The determination of anisotropic part of $^{13}C$ hyperfine tensors from experimental 1D CHEESY-detected NMR spectra (Fig. 7b, top of the main text) is rather uncertain. Therefore, we use the DFT-calculated anisotropic part $a_{dip}$ (1) = [1.26 0.54 -1.80] MHz and $a_{dip}$ (2) = [1.52 0.50 -2.01] MHz for the two $^{13}C$ nuclei in the simulation, together with DFT computed orientations of $^{13}C$ hyperfine tensors. Due to imperfection of DFT calculations for directly coupled $^{13}C$ nuclei, this may cause slight errors in the simulation of the lineshape of $^{13}C$ signals. Nevertheless, the simulation for **1*** appears to predict reasonably the experimental 1D CHEESY-detected NMR spectrum (Fig. 7b of the main text). The experimentally observed decrease of $^{13}C$ signals after the polymerization of $C_2H_4$ is simulated well as a difference between the simulations for labelled complex **1*** and non-labelled complex **1** (Fig. 7b, bottom, of the main text). However, the experimental decrease of $^{13}C$ signal intensity is not as strong as the simulated one. This indicates that not all $^{13}CH_2{}^tBu$ ligands are exchanged to $(CH_2CH_2)_{n-1}(^{13}CH_2{}^tBu)$, It follows that not all the molecules of **1*** become active centers of ethylene polymerization,

likely due to a moderate energy barrier of the first $C_2H_4$ insertion (vide infra). An exact quantification is difficult without a precise knowledge of the full $^{13}C$ hyperfine tensor, which also affects line intensities.

**2.5. α-Agostic C-H interaction and π character of Ti-C bonds of 1 and 1@$Al_2O_{3-700}$**

Estimation of π character in Ti-C bonds for **1** and **1**@$Al_2O_{3-700}$. For both the structures of **1** and **1**@$Al_2O_{3-700}$, the NBO analysis provides the spin-α orbitals, related to the SOMO of these complexes (see Fig. 8 of the main text), together with the lone pairs (LP) on α-C atoms of $CH_2{}^tBu$ ligands instead of Ti-C bonding orbitals (BD). This indicates that the Ti-C bonds in **1** and **1**@$Al_2O_{3-700}$ are strongly polarized, having their electron density mostly on the corresponding carbon atoms. The natural orbitals (e.g. SOMO), produced by these calculations, are related to the conventional molecular orbitals of **1** and **1**@$Al_2O_{3-700}$ and shown on Fig. 8 of the main text. In order to estimate the π character in these bonds, however, the bonding orbitals are required by the program GenNBO[19] to calculate the deviations of corresponding NHO on carbon atoms from the Ti-C bond. To estimate this deviation, separate NBO calculations were performed, where GenNBO was forced to create a bonding set of orbitals for the Ti-C bonds in **1** and **1**@$Al_2O_{3-700}$ ($CHOOSE keyword in GenNBO convention). For all the other studied structures, there was no need of such restrictions, as the Ti-C bonds were represented correctly as BD orbitals in NBO analyses.

**2.6. Olefin Polymerization Pathways of 1 and 1@$Al_2O_{3-700}$**

Structure of complex **1**$^+$. The structure of the model Ti(IV) complex $[Ti(nacnac)(CH_2{}^tBu)]^+$ was obtained from a previously optimized structure of **1** (see Fig. 4a of the main text) through a geometry optimization with the same parameters, as the ones used for the optimization of **1**, except for the charge and multiplicity values (both set to 1).

TS optimizations. All the transition state (TS) optimizations were performed for structures sufficiently close to the expected TS geometries. For the TS of ethylene insertion for **1**, **1**$^+$ and **1**@$Al_2O_{3-700}$, the structures, corresponding to the first ethylene insertion into the Ti-C bonds (i.e. $Ti(nacnac)(CH_2{}^tBu)((CH_2)_3(CH_2{}^tBu))$, $[Ti(nacnac)(CH_2{}^tBu)((CH_2)_3(CH_2{}^tBu))]^+$ and $Ti(nacnac)(OAl_s)((CH_2)_3(CH_2{}^tBu))$, respectively,) were optimized in a preliminary step. The RIJCOSX approsimation[16] with the def2-TZVPP/J auxiliary basis set[17] was used for these optimizations. Furthermore, the γ-C atoms of $(CH_2)_3(CH_2{}^tBu)$ ligands of the obtained structures were approached to the Ti center *via* relaxed surface scans with the step of 0.005 Å. For the TS of α-H abstraction for **1** and **1**$^+$, the α-H atom of one of $CH_2{}^tBu$ ligands, which appears the closest to the α-C atom of the other $CH_2{}^tBu$ ligand in the optimized structures of **1** and **1***, was approached to the indicated α-C atom *via* the relaxed surface scan with the step of 0.005 Å. The TS optimizations were further performed for the structures, corresponding to the maxima of potential energies of the relaxed surface scans, in two steps (with and without RIJCOSX approximation) as described in Materials and Methods part.

All the obtained TS structures are represented in Fig. S13.

**Figure S13.** a) TS of ethylene insertion for the complex **1**. b) TS of ethylene insertion for the complex **1**@$Al_2O_{3-700}$. c) TS of ethylene insertion for the complex **1**$^+$. d) TS of α-H abstraction for the complex **1**. e) TS of α-H abstraction for the complex **1**$^+$. For the TS of ethylene insertion, the elongated Ti-C bond, in which the insertion occurs, is marked with red asterisk.

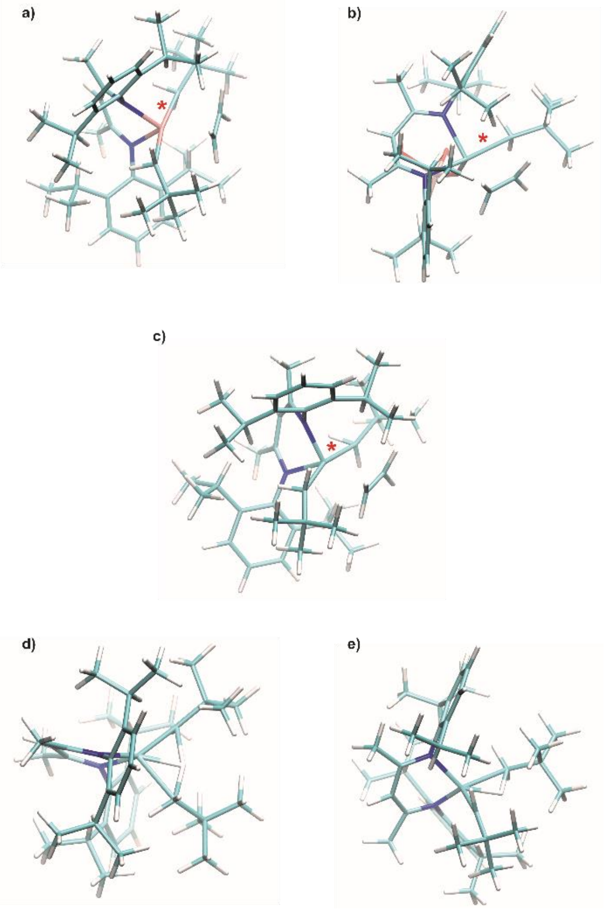


All the obtained TS structures were verified to be the saddlepoints of the corresponding reactions by the presence of a single imaginary frequency after the numerical frequencies calculations. These frequencies are -128.79 cm$^{-1}$, -215.92 cm$^{-1}$ and -187.06 cm$^{-1}$ for the TS of ethylene insertion for **1**, **1**$^+$ and **1**@$Al_2O_{3\text{-}700}$, respectively, and -1283.59 cm$^{-1}$ and -1289.11 cm$^{-1}$ for the TS of α-H abstraction for **1** and **1**$^+$, respectively. For all the TS of ethylene insertion, the quasi-Newton geometry optimizations (step qn; inHess unity in %geom block in ORCA convention) at the terminal structures of corresponding imaginary modes resulted in ethylene insertion for the one side of the modes and to formation of π-ethylene complexes for the other side of the imaginary modes. For all the TS of α-H abstraction, the same procedure results in the initial structures of **1** and **1**$^+$ for the one side of the mode and in the corresponding Ti alkylidenes, together with neopentane, for the other side. These results are valid for both steps of TS optimization at the different levels of theory.

Structures of π-ethylene complexes. All π-ethylene complexes **1**$\cdots C_2H_4$, **1**@$Al_2O_{3\text{-}700}\cdots C_2H_4$ and **1**$^+\cdots C_2H_4$ were obtained after the verification of the TS saddle points. The quasi-Newton optimizations for the one side of the saddle point of imaginary modes at the first step of TS calculations (RIJCOSX approximation[15]) resulted in structures similar to the ones expected for the π-ethylene complexes. These structures were further optimized in two steps (see Materials and Methods part) to obtain the explicit structures of the π-ethylene complexes (Fig. 9 of the main text). While being close by structure to the corresponding TS (although having slightly shortened Ti-C bonds and elongated Ti–$C_2H_4$ distances compared to the TS structures), these complexes were proven by the absence of imaginary frequencies to be true minima of potential energy surfaces within the selected computational method.

Calculation of thermodynamic parameters. Enthalpies (H) and Gibbs free energies (G) were calculated for all obtained structures, using numerical frequencies calculations in ORCA 3.[7] For all the TS for the α-H abstraction, the corresponding TS barriers $\Delta H^{\ddagger}_{298}$ and $\Delta G^{\ddagger}_{298}$ were calculated in comparison with initial optimized structures of **1** and **1**$^+$. For all the TS for ethylene insertion, the corresponding TS barriers $\Delta H^{\ddagger}_{298}$ and $\Delta G^{\ddagger}_{298}$ were calculated in two ways: either with respect to the sum of enthalpies and free energies of initial reagents, namely **1**, **1**$^+$ and **1**@$Al_2O_{3\text{-}700}$ with ethylene, or with respect to the formation energies $\Delta H^{\ddagger}_{298}$ and $\Delta G^{\ddagger}_{298}$ of corresponding π-ethylene complexes **1**$\cdots C_2H_4$, and **1**$^+\cdots C_2H_4$ and **1**@$Al_2O_{3\text{-}700}\cdots C_2H_4$. The formation energies $\Delta H^{0}_{298}$ and $\Delta G^{0}_{298}$ of all the π-ethylene complexes were calculated with respect to the sum of enthalpies and free energies of initial reagents, namely **1**, **1**$^+$ and **1**@$Al_2O_{3\text{-}700}$ with ethylene. In order to calculate thermodynamic parameters of ethylene, the $C_2H_4$ molecule was optimized with the same parameters, as used elsewhere. For comparison among molecular complexes, the COSMO model[15] was used, while not using it for all the grafted models. Note that no basis set superposition errors (BSSE) were considered, which may result in errors of ca. 3 kcal/mol for the comparison of thermodynamic parameters between systems with different number of atoms with the triple-ζ basis sets.[25] For this reason, we didn't compare the $\Delta G^{\ddagger}_{298}$ parameters for α-H abstraction and ethylene insertion reactions for complex **1**, which appear to be different just by 2.7 kcal/mol. For the same two reactions for complex **1**$^+$, the calculated difference in $\Delta G^{\ddagger}_{298}$ is larger than 3 kcal/mol, which allows their comparison. For the TS of ethylene insertion for **1** and **1**@$Al_2O_{3\text{-}700}$, differences in both $\Delta H^{\ddagger}_{298}$ and $\Delta G^{\ddagger}_{298}$ are larger than 3 kcal/mol, which allows their comparison as well.

**2.7. Back donation from the unpaired electron orbital and the enhanced π character in Ti-C bonds of the π-ethylene complexes support polymerization mechanism**

NBO analysis for **1**···$C_2H_4$ and **1**$^{+}$···$C_2H_4$. For both the molecular π-ethylene complexes, namely $d^1$ **1**···$C_2H_4$ and $d^0$ **1**$^{+}$···$C_2H_4$, a back donation to $\pi^*(C_2H_4)$ orbital was revealed by NBO analysis; being zero for the free ethylene molecule, the occupancy of the corresponding natural orbital orbital increases for **1**$^{+}$···$C_2H_4$ and further increases for **1**···$C_2H_4$ (Table S1).

**Table S1.** Occupancies of the spin-α natural orbitals, corresponding to $\pi^*(C_2H_4)$ orbitals of the studied molecular complexes.

| | Occupancy (max. 1) |
|---|---|
| Free $C_2H_4$ | 0.00000 |
| **1**$^{+}$···$C_2H_4$ | 0.06775 |
| **1**···$C_2H_4$ | 0.10328 |

The obtained occupancies indicate that the degree of back donation is much stronger for the $d^1$ complex **1**···$C_2H_4$ than for the $d^0$ complex **1**$^{+}$···$C_2H_4$, which is explained by the back donation of unpaired electron from SOMO of initial complex **1**. For both complexes **1**···$C_2H_4$ and **1**$^{+}$···$C_2H_4$, the back donation leads to stabilization of the complexes. The energy of such stabilization can be determined with NBO energetic analysis by the deletion of the natural orbitals (both spin-α and spin-β), corresponding to $\pi^*(C_2H_4)$ of **1**···$C_2H_4$ and **1**$^{+}$···$C_2H_4$. After the deletion, the SCF energy growth was found to be 23.134 kcal/mol for **1**$^{+}$···$C_2H_4$, and 30.044 kcal/mol for **1**···$C_2H_4$, indicating the corresponding stabilization energies. Assuming that the back donation, which is not related to the unpaired electron of SOMO (e.g. the electron density, donated from another ligands), has the same degree for both **1**···$C_2H_4$ and **1**$^{+}$···$C_2H_4$, the pure stabilization, caused by the back donation of unpaired electron density, was found to be 6.91 kcal/mol.

NBO analysis of **1**@$Al_2O_{3\text{-}700}$···$C_2H_4$. An even stronger degree of back donation is found for the **1**@$Al_2O_{3\text{-}700}$···$C_2H_4$ complex. The NBO analysis indicates a strong delocalization of unpaired electron between the natural orbitals, produced by NBO program[18] for **1**@$Al_2O_{3\text{-}700}$···$C_2H_4$ complex (Table S2).

**Table S2.** Important spin-α natural orbitals, produced by NBO analysis for the **1**@$Al_2O_{3\text{-}700}$···$C_2H_4$ complex. The natural orbitals are marked within NBO program[18] internal convention.

| Natural orbital | Occupancy (max. 1) |
|---|---|
| BD (Ti47 – C79) | 0.91693 |
| BD* (Ti47 – C79) | 0.36183 |
| LP (C80) | 0.55276 |
| BD (Ti47 – C81) | 0.95654 |
| BD* (Ti47 – C81) | 0.13840 |

In fact, NBO analysis indicates a bonding interaction between the Ti center and one of the carbon atoms of $C_2H_4$; the related bonding set of orbitals BD (Ti47 – C79) and BD* (Ti47 – C79) is shown on Fig. S14, a & b. The corresponding bond order, determined as a difference between the occupancies of bonding (BD) and antibonding (BD*) spin-α natural orbitals, is equal to 0.555. The half-occupied lone pair LP (C80) corresponds to the $\pi^*(C_2H_4)$ orbital, being polarized towards one of the carbon atoms of $C_2H_4$, as revealed by its spatial distribution (Fig. S14, c). Also the population of BD* (Ti47 – C81), being related to $\sigma^*$(Ti-C), is observed (Table S2).

**Figure S14.** a) Natural spin-α orbital, marked as BD (Ti47 – C79). b) Natural spin-α orbital, marked as BD* (Ti47 – C79). c) Natural spin-α orbital, marked as LP (C80).

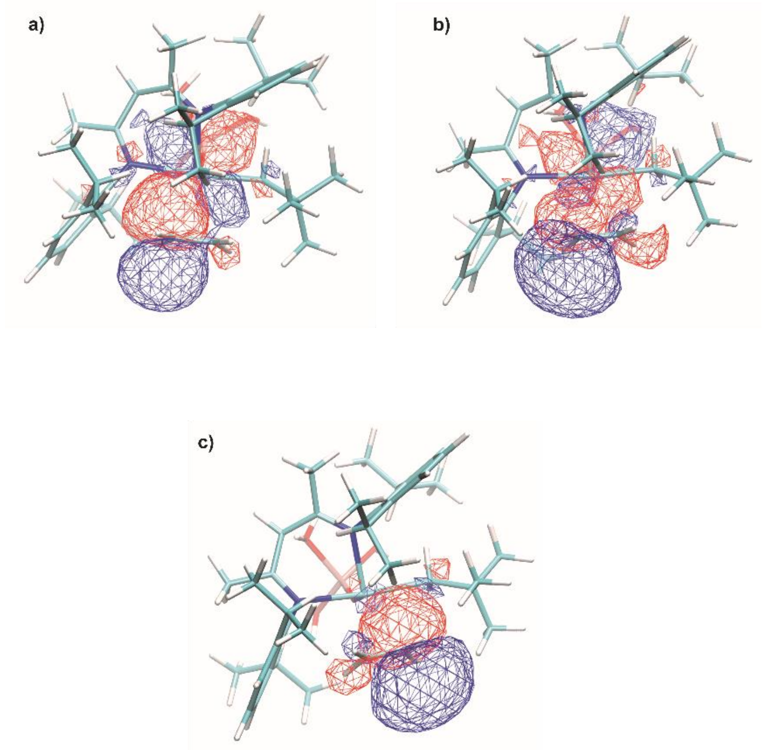


Together with the calculated spin density distribution (see Fig. 10b of the main text), the NBO analysis, being one possible way of representation of the electronic structure of **1**@$Al_2O_{3\text{-}700}$···$C_2H_4$ complex, indicates a strong delocalization of the unpaired electron between SOMO (Ti), $\pi^*(C_2H_4)$ and $\sigma^*$(Ti-C). Note that both the π-ethylene complexes are close by their structures and energies to the TS of ethylene insertion; while approaching the TS, the degree of delocalization of the unpaired electron further enhances (see Fig. 10, b & d of the main text). This delocalization leads to a weakening of $\pi(C_2H_4)$ and σ(Ti-C) bonds due to the population of corresponding antibonding orbitals (Table S2), which further facilitates the process of olefin insertion. Hence, the delocalization of unpaired electron in $d^1$ π-ethylene complexes and in the TS of ethylene insertion (see Scheme 2 of the main text), together with a strong π character in the metal-carbon bonds, appears to be an energetically more favorable mechanism of olefin insertion, which may be defined as “augmented” Cossee-Arlman mechanism.

## References


1 S. Stoll and A. Schweiger, *J. Magn. Reson.*, 2006, **178**, 42 – 55.
2 Y. Polyhach, E. Bordignon, R. Tschaggelar, S. Gandra, A. Godt and G. Jeschke, *Phys. Chem. Chem. Phys.*, 2012, **14**, 10762 – 10773.
3 A. Doll and G. Jeschke, *J. Magn. Reson.*, 2017, **280**, 46 – 62.
4 N. Wili, G. Jeschke, *J. Magn. Reson.*, 2018, **289**, 26 – 34.
5 L. F. Ibáñez, Hyscorean, ETH Zürich, Zürich, Switzerland, 2018, https://epr.ethz.ch/software.html
6 N. Cox, A. Nalepa, W. Lubitz and A. Savitsky, *J. Magn. Reson.*, 2017, **280**, 63 – 78.
7 N. Wili, S. Richert, B. Limburg, S. J. Clarke, H. L. Anderson, C. R. Timmel and G. Jeschke, *Phys. Chem. Chem. Phys.*, 2019**, 21**, 11676 – 11688.
8 F. Neese, *Wiley Interdisciplinary Reviews: Computational Molecular Science*, 2012, **2**, 73 – 78.
9 J. P. Perdew and M. Ernzerhof, *J. Chem. Phys*., 1996, **105**, 9982 – 9985; C. Adamo and V. Barone, *J. Chem. Phys*., 1991, **110**, 6158 – 6170.
10 F. Weigend and R. Ahlrichs, *Phys. Chem. Chem. Phys.*, 2005**, 7**, 3297 – 3305.
11 N. Rega, M. Cossi and V. Barone, *J. Chem. Phys*., 1996, **105**, 11060 – 11067.
12 W. Humphrey, A. Dalke and K. Schulten, *J. Mol. Graph*., 1996, **14**, 33 – 38.
13 S. Gohr, P. Hrobárik, M. Repiský, S. Komorovský, K. Ruud and M. Kaupp, *J. Phys. Chem*. *A*, 2015, **119**, 12892 – 12905.
14 S. Grimme, J. Antony, S. Ehrlich and H. Krieg, *J. Chem. Phys*., 2010, **132**, 105104; S. Grimme, S. Ehrlich and L. Goerigk, *J. Comput. Chem*., 2011, **32**, 1456 – 1465.
15 A. Klamt and G. Schüürmann, *J. Chem. Soc. Perkin Trans 2*, 1993, 799 – 805.
16 F. Neese, F. Wennmohs, A. Hansen and U. Becker, *Chem. Phys.*, 2009, **356**, 98 – 109.
17 F. Weigend, *Phys. Chem. Chem. Phys.*, 2006, **8**, 1057 – 1065.
18 ORCA manual, https://cec.mpg.de/fileadmin/media/Forschung/ORCA/orca_manual_4_0_1.pdf, (accessed August 2020).
19 F. Weinhold, The Natural Bond Orbital (NBO) program NBO 7.0, University of Wisconsin, Madison (US), 2018.
20 F. Neese, *Wiley Interdisciplinary Reviews: Computational Molecular Science*, 2017, **8**, e1327.
21 NBO 7.0 Program Manual, https://nbo6.chem.wisc.edu/nboman.pdf, (accessed August 2020).
22 E.D. Glendening, C. R. Landis and F. Weinhold, *WIREs Comput. Mol. Sci.*, 2012, **2**, 1 – 42.
23 C. P. Gordon, S. Shirase, K. Yamamoto, R. A. Andersen, O. Eisenstein and C. Copéret, *Proc. Natl. Acad. Sci.*, 2018, **115**, 5867 – 5876.
24 F. Basuli, B. C. Bailey, J. Tomaszewski, J. C. Huffman and D. J. Mindiola, *J. Am. Chem. Soc.*, 2003**, 125**, 6052 – 6053.
25 E. Miliordos and S. S. Xantheas, *J. Chem. Phys.*, 2015, **142**, 094311.
